# Dissecting the roles of MBD2 isoforms and domains in regulating NuRD complex function during cellular differentiation

**DOI:** 10.1038/s41467-023-39551-w

**Published:** 2023-06-29

**Authors:** Nina Schmolka, Ino D. Karemaker, Richard Cardoso da Silva, Davide C. Recchia, Vincent Spegg, Jahnavi Bhaskaran, Michael Teske, Nathalie P. de Wagenaar, Matthias Altmeyer, Tuncay Baubec

**Affiliations:** 1 Department of Molecular Mechanisms of Disease, University of Zurich, Zurich, Switzerland; 2 Genome Biology and Epigenetics, Institute of Biodynamics and Biocomplexity, Department of Biology, Utrecht University, Utrecht, The Netherlands; 3 Molecular Life Science PhD Program of the Life Science Zurich Graduate School, University of Zurich and ETH Zurich, Zurich, Switzerland; 4 Present Address: Institute of Experimental Immunology, University of Zurich, Zurich, Switzerland; 5 Present Address: MRC London Institute of Medical Sciences, London, UK

**Keywords:** Chromatin remodelling, Stem-cell differentiation

## Abstract

The Nucleosome Remodeling and Deacetylation (NuRD) complex is a crucial regulator of cellular differentiation. Two members of the Methyl-CpG-binding domain (MBD) protein family, MBD2 and MBD3, are known to be integral, but mutually exclusive subunits of the NuRD complex. Several MBD2 and MBD3 isoforms are present in mammalian cells, resulting in distinct MBD-NuRD complexes. Whether these different complexes serve distinct functional activities during differentiation is not fully explored. Based on the essential role of MBD3 in lineage commitment, we systematically investigated a diverse set of MBD2 and MBD3 variants for their potential to rescue the differentiation block observed for mouse embryonic stem cells (ESCs) lacking MBD3. While MBD3 is indeed crucial for ESC differentiation to neuronal cells, it functions independently of its MBD domain. We further identify that MBD2 isoforms can replace MBD3 during lineage commitment, however with different potential. Full-length MBD2a only partially rescues the differentiation block, while MBD2b, an isoform lacking an N-terminal GR-rich repeat, fully rescues the *Mbd3* KO phenotype. In case of MBD2a, we further show that removing the methylated DNA binding capacity or the GR-rich repeat enables full redundancy to MBD3, highlighting the synergistic requirements for these domains in diversifying NuRD complex function.

## Abstract (web summary)

MBD proteins are integral subunits of the NuRD complex, a crucial regulator of cellular differentiation. Here the authors systematically dissect which MBD protein domains and isoforms are essential for neuronal differentiation.

## Introduction

Cellular differentiation entails establishment of new cell identities through changes in transcriptional programs. Multiple components involving external stimuli, transcription factors and chromatin modifications play important roles in orchestrating gene expression during developmental transitions. Chromatin remodeling complexes are key components of this process, enabling the change of chromatin structure and the accessibility of specific genomic sites^1^. The Nucleosome Remodeling and Deacetylation (NuRD) complex is an abundant and highly conserved complex, regulating pluripotency, cell fate transitions and differentiation in many different organisms and developmental contexts^2–6^. The multiprotein complex combines two enzymatic activities: lysine deacetylation mediated by Histone Deacetylase (HDAC) 1 and 2 proteins, and ATPase-dependent nucleosome remodeling by Chromodomain Helicase DNA binding protein (CHD) 3 or 4^7–9^. Additional complex partners are the histone chaperone proteins RBBP4 and 7, the zinc-finger proteins GATAD2a or GATAD2b, two MTA proteins (MTA1, MTA2, and/or MTA3) and CDK2AP1^10^. Additionally, the methyl-CpG binding protein family members MBD2 or MBD3 are essential but mutually exclusive NuRD complex members, therefore assembling distinct MBD2-NuRD or MBD3-NuRD complexes^4,11^. Recent structural and biochemical data supports the notion that the MBD2 and MBD3 proteins function as a link between the MTA:HDAC:RBBP core and the peripheral GATAD2:CHD:CDK2AP remodeling module^12–14^. Absence of MBD2 or MBD3 therefore disrupts NuRD complex functionality. In addition, replacement of MBD2 or MBD3 through PWWP2A results in a distinct complex lacking the remodeling module, also called NuDe complex^15–17^. In vivo, MBD2 seems dispensable for normal mouse development as *Mbd2* KO mice display only minor phenotypes but are viable and fertile^4^. In contrast, MBD3 is required to exit pluripotency and essential for early mammalian development reflected by lethality of *Mbd3* KO mouse embryos^4,18–20^.

MBD2 and MBD3 are closely related proteins that share almost 80% homology outside of the MBD domain and arose by gene duplication from an ancestral MBD2/3 gene that is present in some metazoans^4,11,21^. MBD2 and MBD3 contain an MBD and a coiled-coil domain (CC) separated by a disordered protein region, with the latter two being important for protein-protein interaction with the NuRD complex^22–24^. Whereas the MBD domain of MBD2 shows high affinity for methylated DNA, the MBD3-MBD domain lacks four conserved amino acids required for the recognition of methyl-CpG^25^. In addition, MBD2 contains an N-terminal glycine-arginine (GR) rich stretch that has been implicated in increasing DNA methylation affinity and interactions with the NuRD complex^11,26^. Differential inclusion of these domains results in various MBD2 and MBD3 isoforms, some with cell type or tissue-specific expression^18,27–29^. Three MBD3 isoforms are present in mouse ESCs: The full-length MBD3a isoform, MBD3b with a truncated MBD domain and MBD3c lacking the MBD domain^18^. MBD2 also contains three isoforms: the full-length MBD2a, MBD2b lacking the N-terminal GR repeat, and MBD2t lacking the C-terminal CC domain. Based on the presence of either MBD2 or MBD3 in the NuRD complex, MBD2-NuRD and MBD3-NuRD are considered to have distinct functional roles during early development. It is speculated that this is mainly due to their differential binding affinity to methylated DNA by the MBD proteins and recruitment to distinct genomic sites. The tissue-specific presence of MBD2 or MBD3 isoforms is expected to further increase the complexity of NuRD complex function. Still, little is known about the direct requirement of the individual MBD2 and MBD3 domains for NuRD complex activity during cellular differentiation. Furthermore, differential and overlapping expression levels of MBD2 and MBD3 isoforms in different cellular contexts have convoluted our current understanding about the roles of these different NuRD complexes, requiring further investigation.

Here, we took a systematic approach to dissect the functionality of different NuRD complex compositions during neuronal commitment and terminal differentiation through controlling the expression of MBD2- or MBD3-isoforms. Towards this, we combined neuronal differentiation of engineered murine ESCs with FACS-based measurements of cell identity and transcriptional profiling. In our approach, successful lineage commitment is a direct measurement of a functional NuRD complex reconstituted with specific MBD isoforms. We show that MBD3 is indeed the critical NuRD complex member allowing neuronal differentiation, but this function is independent of its MBD domain. Additionally, full-length MBD2a can partially compensate for MBD3 function. In absence of the GR-stretch or DNA methylation binding affinity, this ability is further elevated to fully compensate for the absence of MBD3, indicating that these protein regions prevent a complete redundancy to MBD3. We show that the MBD2 GR-stretch and MBD domain act synergistically to sequester MBD2-NuRD to chromocenters.

## Results

### Quantitative readout for NuRD complex function

**Fig. 1:**
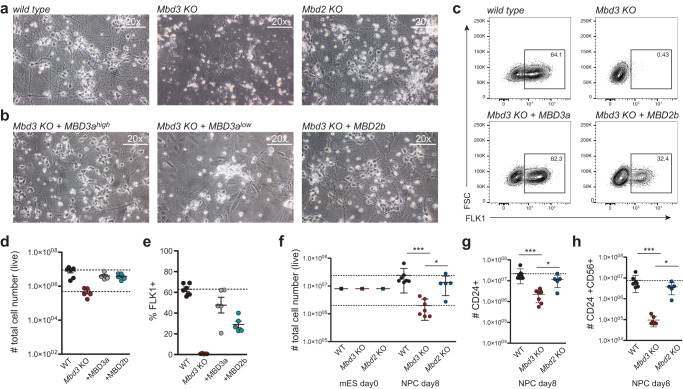
Neuronal differentiation in absence of MBD2 and MBD3. **a** Microscopy images of in vitro derived neurons from WT, *Mbd3* KO, and *Mbd2* KO ESCs shown at 20× magnification. Same results were obtained from 3-5 independent replicates. **b** Microscopy images of in vitro derived neurons from *Mbd3* KO ESCs stably expressing MBD3a (high) or MBD2b under a strong promoter (CAG) or MBD3a (low) under a weaker promoter (CMV). Shown at 20× magnification. Same results were obtained from 3 independent replicates. **c** Representative flow cytometry analysis of FLK1 surface expression and flow cytometry analysis. **d**, **e** Number of live cells (**d**) and percentage of FLK1+ cells (**e**) in EB cultures of WT, *Mbd3* KO, and *Mbd3* KO cell lines expressing MBD3a or MBD2b. **f**–**h** Flow cytometry analysis showing number of live cells (WT to *Mbd3* KO p = 0.0006, *Mbd3* KO to *Mbd2* KO p = 0.0147) (**f**), CD24+ cells (WT to *Mbd3* KO p = 0.0002, *Mbd3* KO to *Mbd2* KO p **=** 0.0451) (**g**) and CD24 + CD56+ cells (WT to *Mbd3* KO p = 0.0002, *Mbd3* KO to *Mbd2* KO p = 0.0186) (**h**). Each data point (in **d**–**h**) represents individually generated cell lines. For each cell line 3 independent clones were analyzed, n = 2 biological replicates. Error bars represent mean +/− SD. p-values were calculated using an unpaired, two-tailed *t* test (Mann-Whitney). Source data for (**d**–**h**) are provided as a Source Data file.

To investigate the distinct roles of MBD2 and MBD3 during lineage commitment, we employed a well-established in vitro differentiation system of ESCs towards homogenous populations of neural progenitor cells (NPC) and terminal neurons (TN)^30^ (Supplementary Fig. 1a). We first tested the suitability of this setup by differentiating TNs from individual *Mbd2* and *Mbd3* knock-out (KO) ESC cell clones that were generated in the same genetic background using CRISPR-Cas9 (Supplementary Fig. 1b–c). As expected, and in line with previous reports^18^, *Mbd3* KO ESCs were not able to form TNs, while *Mbd2* KO ESCs successfully differentiated towards terminal neurons (Fig. 1a and Supplementary Fig. 1d). Next, we assessed the capacity of MBD3a, the longest MBD3 isoform, to rescue the neuronal differentiation phenotype of *Mbd3* KO cells. Towards this, we expressed MBD3 at two different levels from a defined genomic site in the mouse ESC genome (Supplementary Fig. 1e and f)^24^. Expression of the MBD3a isoform in *Mbd3* KO ESCs fully rescued the differentiation block and TNs were formed similar to WT control cells (Fig. 1b). However, this was only observed when we expressed MBD3 at high levels, while MBD3 expression at lower levels was not sufficient to fully rescue the differentiation block (Fig. 1b). Next, we wanted to test the capacity of MBD2 to rescue the observed phenotype and assess a potential redundancy between MBD3 and MBD2 during neuronal differentiation. Towards this, we chose the shorter MBD2 isoform, MBD2b, which is very similar to MBD3a in terms of domain composition, and expressed it from the same genomic integration site using a strong promoter (Supplementary Fig. 1e). To our surprise, MBD2b could fully rescue the *Mbd3* KO phenotype, leading to fully differentiated TNs (Fig. 1b). We additionally tested our observation with another well-established ES cell differentiation model via embryoid bodies towards FLK1^+^ mesoderm^31–33^. Similar to the neuronal differentiation, *Mbd3* KO ES cells showed a complete block in mesoderm differentiation (Fig. 1c–e). Re-expression of MBD3a fully rescued this differentiation block, and similar to the neuronal differentiation, MBD2b expression enabled *Mbd3* KO ES cells to form FLK1^+^ mesoderm cells (Fig. 1c–e).

Based on these initial observations, we wanted to investigate the contribution of the individual MBD2 and MBD3 isoforms towards NuRD complex function using a quantitative and systematic, differentiation-based readout. We explored published microarray expression data^34^ of several surface proteins at consecutive neuronal differentiation stages (ESC, cell aggregate formation (CA) day 4, NPC day 8 and TN day 2 and day 4, respectively) and identified the two neuronal surface proteins, CD24a (CD24) and CD56 (also known as NCAM1), as significantly up regulated at the NPC and TN stage (Supplementary Fig. 2a). To test if CD24 and CD56 indicate successful neuronal lineage commitment, we sorted different NPC populations by FACS from differentiated WT ESCs. As expected, CD24/CD56 double negative NPCs were not able to form TNs, whereas both cells expressing CD24 alone and with CD56 formed TNs (Supplementary Fig. 2b, c). In this study, we subsequently used this FACS-based readout of CD24 and CD56 surface expression on NPCs as a measure of successful ESC lineage commitment in addition to the total amount of live cells generated at progenitor stage and morphological assessment of fully differentiated neurons.

To underline the suitability of this FACS readout, we repeated the differentiation of the *Mbd2* and *Mbd3* KO cell lines together with wild type cells and measured CD24 and CD56 levels at the NPC stage (Supplementary Fig. 2d). Whereas uncommitted ESCs do not express CD24 and CD56 in all three tested lines (WT, *Mbd2* KO, and *Mbd3* KO), NPCs derived from both WT cells or *Mbd2* KO generated comparable numbers of live cells, CD24^+^ single, and CD24^+^CD56^+^ double-positive cells, indicating successful neuronal commitment (Fig. 1f–h). This was in stark contrast to the *Mbd3* KO ESCs that showed a >10-fold reduction of total number of live cells, CD24^+^ single and CD24^+^CD56^+^ double-positive cells (Fig. 1f–h). Additionally, we detected a significant increase in the frequency of uncommitted CD24^−^CD56^−^ cells in *Mbd3* KO cells, when compared to WT (12% in WT vs. 38% in *Mbd3* KO) (Supplementary Fig. 2e).

### The MBD domain of MBD3 is dispensable for neuronal differentiation

**Fig. 2:**
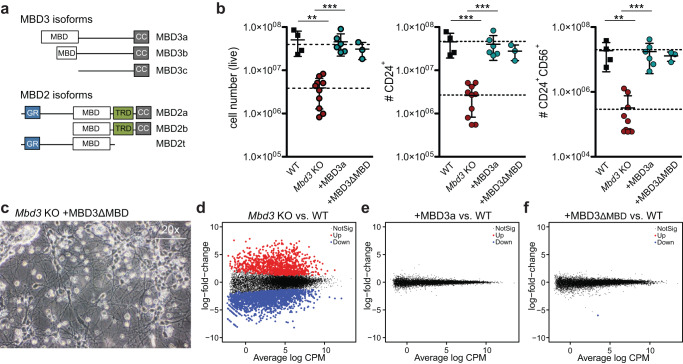
The MBD domain of MBD3 is dispensable for ESC neuronal lineage differentiation. **a** Schematic of MBD3 and MBD2 isoforms present in ESCs. MBD: MBD domain, cc: coil-coiled domain, GR: glycine- arginine rich repeat, TRD: transcriptional repressor domain. **b** Flow cytometry analysis indicating (from left to right) the number of live cells (WT to *Mbd3* KO p = 0.0020, *Mbd3* KO to +MBD3a p = 0.0001), CD24+ (WT to *Mbd3* KO p = 0.0001, *Mbd3* KO to +MBD3a p = 0.0002), or CD24 + CD56 + NPC cells (WT to *Mbd3* KO p = 0.0020, *Mbd3* KO to +MBD3a p = 0.0002) at day 8 of WT, *Mbd3* KO, and *Mbd3* KO stably expressing MBD3a or MBD3ΔMBD variants. Each data point represents an individual cell line. For each cell line 3 independent clones were analyzed, n = 2 biological replicates. Error bars represent mean +/− SD. p values were calculated using an unpaired, two-tailed t test (Mann-Whitney). Source data is provided as a Source Data file. **c** Microscopy images of in vitro derived neurons from *Mbd3* KO stably expressing MBD3ΔMBD at 20× magnification. Identical results were obtained from 3 independent replicates. **d–f** MA-plots showing differential gene expression between *Mbd3* KO and WT NPCs (**d**), *Mbd3* KO + MBD3a vs WT NPCs (**e**), and *Mbd3* KO + MBD3ΔMBD vs WT NPCs (**f**). Red and blue dots indicate genes with significant changes in gene expression (edgeR, log2FC > 1 | < −1 and adjusted p-value < 0.05).

To systematically test the functional role of different isoforms and domains in regulating neuronal lineage commitment, we repeated the experiments outlined above with additional MBD2 and MBD3 variants expressed at comparable levels from the same genomic integration site in *Mbd3* KO ESCs (Fig. 2a). In addition to the full-length MBD3a isoform that contains the complete MBD domain, two additional MBD3 isoforms are present in ESCs: one with a truncated (MBD3b) and the other with a complete lack (MBD3c) of the N-terminal MBD domain (Fig. 2a and Supplementary Fig. 2f)^18,27^. Previous reports suggest that all three isoforms are equally capable of promoting lineage commitment^18,35^. To evaluate the role of the MBD domain of MBD3 in neuronal differentiation, we generated *Mbd3* KO ESCs expressing a MBD3 protein lacking the entire MBD domain (MBD3ΔMBD). Similar to the MBD3a full-length variant, the MBD3ΔMBD construct completely rescued the differentiation block of the *Mbd3* KO ESCs at the progenitor (Fig. 2b) and neuron (Fig. 2c) stage, in agreement with previous results using MBD3c^35^.

To obtain a more detailed view of the molecular response following re-expression of wild type MBD3 and MBD3ΔMBD in *Mbd3* KO cells, we performed RNA-seq at the neuronal progenitor cell (NPC) stage and investigated the resulting changes in gene expression. Differential gene expression analysis between WT and *Mbd3* KO cells revealed drastic changes in gene expression with 1821 genes up-regulated and 1102 genes down-regulated in the *Mbd3* KO NPCs (Fig. 2d). As expected, transcriptional changes resulted in downregulation of genes associated with GO terms “neuronal differentiation”, while upregulated genes were associated with GO terms “immune response” (Supplementary Fig. 3a–c). In accordance with the results above, these changes were completely reverted upon reintroduction of the wild type MBD3a or MBD3ΔMBD proteins, indicating a full rescue of the *Mbd3* KO phenotype, irrespective of MBD domain presence (Fig. 2e, f).

### MBD2 isoforms vary in their ability to rescue the *Mbd3* KO differentiation block

**Fig. 3:**
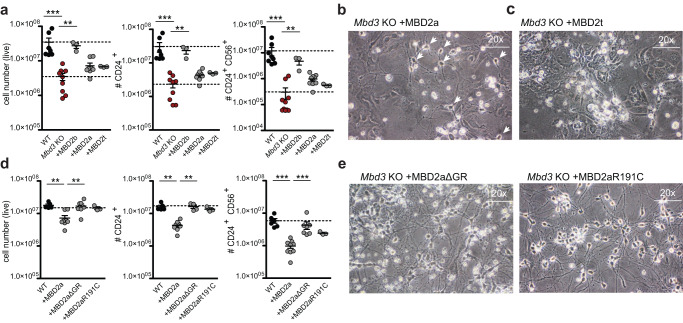
Full-length MBD2a partially rescues the differentiation block in *Mbd3* KO ESCs. **a** Flow cytometry analysis indicating the number of live cells (left, WT to *Mbd3* KO p = 0.0003, *Mbd3* KO to +MBD2b p = 0.0091), CD24+ (middle, WT to *Mbd3* KO p = 0.0002, *Mbd3* KO to +MBD2b p = 0.0091), and CD24 + CD56+ (right, WT to *Mbd3* KO p = 0.0001, *Mbd3* KO to +MBD2b p = 0.0070) NPCs at day8 of neuronal differentiation of WT, *Mbd3* KO, or *Mbd3* KO stably expressing MBD2b, MBD2a or MBD2t. Each data point represents individual cell lines. For each cell line 3 independent clones were analyzed (biological replicates), except for MBD2t, where three technical replicates of one clone were analyzed. Error bars represent mean +/− SD. p values were calculated using an unpaired, two-tailed t test (Mann-Whitney). Source data is provided as a Source Data file. Microscopy images of in vitro derived neurons from *Mbd3* KO stably expressing MBD2a (**b**) or MBD2t (**c**) at 20× magnification. Similar results were obtained from 3-5 independent replicates. **d** Flow cytometry analysis indicating the number of live cells (left, WT to +MBD2a p = 0.0016, +MBD2a to +MBDaΔGR p = 0.0012), CD24+ (middle, WT to +MBD2a p = 0.0011, +MBD2a to +MBDaΔGR p = 0.0040), and CD24 + CD56+ (right, WT to +MBD2a p = 0.0004, +MBD2a to +MBDaΔGR p = 0.0002) NPCs at day8 of neuronal differentiation of WT or *Mbd3* KO stably expressing MBD2a, MBD2aΔGR or MBD2aR191C. Each data point represents individual cell lines. For each cell line 3 independent clones were analyzed, n = 2 biological replicates. Error bars represent mean +/− SD. p values were calculated using an unpaired, two-tailed t test (Mann-Whitney). Source data is provided as a Source Data file. **e** Microscopy images of in vitro derived neurons from *Mbd3* KO stably expressing MBD2aΔGR or MBD2aR191C at 20× magnification. Similar results were obtained from 3-5 independent replicates.

Following these results, we aimed to analyze how MBD2 isoforms and variants impact NuRD function (Fig. 2a). As initially shown, expression of the MBD2b isoform in *Mbd3* KO ESCs fully rescued the differentiation block to levels observed in WT cells or *Mbd3* KO cells rescued with MBD3a (Fig. 1b). This was further confirmed by FACS in NPCs (Fig. 3a). This shorter MBD2b isoform, lacks an N-terminal stretch, containing a repetitive glycine-arginine (GR) -rich region that is present in the longer MBD2a isoform^27^. To our surprise, expression of the full-length isoform MBD2a could not rescue the defective lineage commitment of *Mbd3* KO ESC. In this case, we only observed a small increase in CD24^+^CD56^+^ NPC cell numbers upon MBD2a expression in the *Mbd3* KO cells (Fig. 3a). This difference between MBD2a and MBD2b is not due to lower expression of MBD2a, since we observe similar levels to MBD3 and MBD2b expression (Supplementary Fig. 4a, b). In addition, no rescue was observed when introducing the truncated isoform MBD2t that lacks a C-terminal coiled coil (CC) domain required for interactions with the GATAD2:CHD module of the NuRD complex^23,24^ (Fig. 3a), suggesting that the full rescue observed for MBD2b requires interactions with the NuRD complex. Despite the low number of CD24^+^ and CD24^+^CD56^+^ double-positive neuronal progenitors in the MBD2a-expressing *Mbd3* KO ESCs, the few surviving cells were able to generate terminal neurons (Fig. 3b). This was in stark contrast to the absence of neurons in *Mbd3* KO or MBD2t-expressing *Mbd3* KO cells (Fig. 3c). Therefore, expression of MBD2a, but not MBD2t, leads to a partial rescue of the neuronal differentiation block of *Mbd3* KO ESCs, indicating that MBD2 isoforms vary in their potential to rescue the lack of MBD3.

### MBD2 MBD domain and repetitive GR stretch serve non-redundant functions

Since the individual MBD2 isoforms vary in their ability to rescue the neuronal differentiation block in *Mbd3* KO ESCs, we wanted to explore the contribution of the different MBD2 domains and their impact on NuRD complex function during neuronal differentiation. MBD2a and MBD2b vary in their N-terminal region. To test if the N-terminal region has an impact on MBD2-NuRD function we introduced an engineered MBD2a variant that only lacks the GR stretch (deletion of 33 AA) (MBD2aΔGR) but retaining the remaining N-terminal part of the protein. Similar to MBD2b, heterologous expression of MBD2aΔGR in *Mbd3* KO ESCs fully restored the neuronal differentiation capacity (Fig. 3d, e). Next, we assessed if the mCpG sensitivity of MBD2 contributes to the partial redundancy. The mCpG binding preference distinguishes the MBD domains of MBD2 and MBD3. To test the impact of mCpG sensitivity of MBD2 in NuRD function, we introduced a mutation in the MBD domain (R191C), which is known to abrogate the MBD domain affinity towards methylated DNA^36^. Expression of MBD2a R191C in MBD3 KO cells rescued the neuronal differentiation block to similar levels observed in WT cells, MBD2aΔGR, and MBD2b add-backs (Fig. 3d, e).

**Fig. 4:**
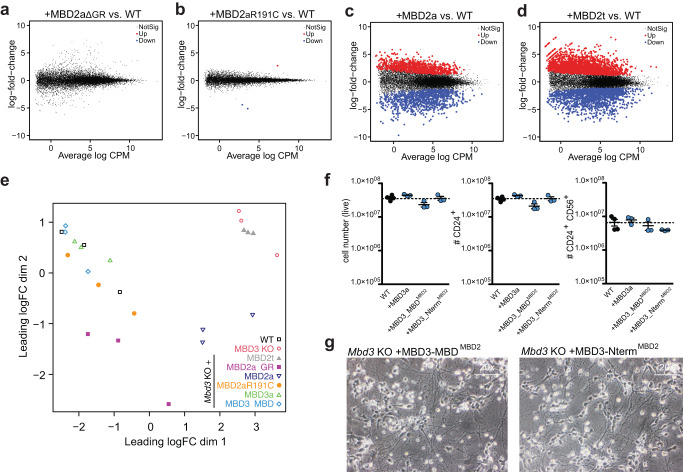
The MBD domain and GR repeat of MBD2 serve non-redundant functions during neuronal differentiation. **a–d** MA-plots showing differential gene expression between *Mbd3* KO+MBD2aΔGR vs. WT NPC (**a**), *Mbd3* KO + MBD2aR191C vs WT NPCs (**b**), *Mbd3* KO + MBD2a vs WT NPCs (**c**), *Mbd3* KO + MBD2t vs WT NPCs (**d**). Red and blue dots indicate genes with significant changes in gene expression (edgeR, log2FC > 1 | < −1 and adjusted p-value < 0.05). **e** Multi-dimensional scaling (MDS) plot indicates the degree of similarity for all RNA-seq datasets obtained from NPCs at day8. Each point represents an individually derived clone except for *Mbd3* KO + MBD2t, where triplicates of one clone were analyzed. **f** Flow cytometry analysis indicating (from left to right) the number of live cells, CD24+, and CD24 + CD56+ NPCs at day 8 of neuronal differentiation of WT or *Mbd3* KO stably expressing MBD3a, MBD3_MBD^MBD2^, or MBD3_Nterm^MBD2^. Each data point represents individual cell lines. For each cell line 3 independent clones were analyzed, n = 1 biological replicate. Source data is provided as a Source Data file. **g** Microscopy images of in vitro derived neurons from *Mbd3* KO stably expressing MBD3_MBD^MBD2^, or MBD3_Nterm^MBD2^at 20x magnification. Similar results were obtained from 3 independent replicates.

These results were further supported by gene expression analysis in neuronal progenitors. Similar to the re-expression of MBD3 proteins, *Mbd3* KO NPCs expressing MBD2aΔGR and MBD2aR191C show no to neglectable gene expression changes (0 up / 0 down, 1up / 2 down-regulated genes, respectively) (Fig. 4a, b). In contrast, cells expressing MBD2a had a high degree of deregulated genes (859 up / 1008 down), and MBD2t (2079 up / 1578 down) (Fig. 4c, d), in line with partial or full failure to rescue differentiation, respectively. Comparing the global gene expression profiles by multidimensional scaling further indicates similarities in gene expression between wild-type NPCs and *Mbd3* KO NPCs expressing MBD3a, MBD3ΔMBD, MBD2aΔGR, and MBD2aR191C (Fig. 4e, Supplementary Fig. 5a, b). The cluster of these cell lines is separated from *Mbd3* KO expressing MBD2t that show a full differentiation block, while the *Mbd3* KO cells expressing MBD2a form an out-group that is nearer to cell lines failing to fully differentiate (Fig. 4e, Supplementary Fig. 5a, b). Focusing on the relevant lineage markers, we observe that cell lines expressing MBD3, MBD3ΔMBD, MBD2aΔGR, MBD2aR191C are able to up-regulate neuronal markers like *Neurog1*, *Neurod4*, and *Pax6* whereas *Mbd3* KO lines that express MBD2a or MBD2t maintain a pluripotent signature with high expression of embryonic stem cell-specific genes like *Pou5f1* (Oct4), *Nanog* and *Klf4*, similar to *Mbd3* KO cells (Supplementary Fig. 5c). Furthermore, by comparing genes differentially expressed in *Mbd3* KO and *Mbd3* KO expressing MBD2a we observe a similar trend in gene expression changes with GO terms “neuronal differentiation” enriched among downregulated genes and “immune response” enriched among upregulated genes (Supplementary Fig. 6a, b). Transcription factor response analysis using ISMARA^37^ reveals that both datasets share co-regulated TF targets, but with *Mbd3* KO showing a stronger response compared to *Mbd3* KO + MBD2a (Supplementary Fig. 6c).

### MBD2 MBD domain or GR stretch alone have no influence on NuRD complex activity

Following these observations, we wanted to explore the mechanism underlying the differential rescue efficiency by the MBD2 variant proteins. Since we initially performed all experiments in an *Mbd2* wild-type background, we first wanted to exclude the possibility of the endogenous MBD2 expression potentially interfering with our measurements. Therefore, we generated a *Mbd2*, *Mbd3* double-KO (DKO) line by targeting exons 2 and 3 of *Mbd3* in a validated *Mbd2* KO ESC line cell (Supplementary Fig. 7a). We next used this DKO cell line to add-back different MBD2 variant proteins and repeated the neuronal differentiation experiments previously performed in *Mbd3* KO cells. We obtained identical results, where we observed that the full-length MBD2a results in partial differentiation rescue, while MBD2aΔGR and MBD2aR191C fully rescue the differentiation block, suggesting that endogenous MBD2 expression did not influence our readout (Supplementary Fig. 7b, c).

The results obtained so far indicate a role for the MBD domain and GR stretch of MBD2 in diversifying NuRD complex function in absence of MBD3. The GR stretch was previously shown to influence mCpG-binding capacity of the MBD domain^11,26^. In line with the lack of redundancy we observed in our rescue experiments, this suggests that both protein parts could act synergistically. To further test this hypothesis, we generated chimeric MBD3 proteins where we replaced either the MBD domain with that of MBD2 (MBD3_MBD^MBD2^), or fused the N-terminal part of MBD2 containing the GR stretch (MBD3_Nterm^MBD2^). The MBD3 KO cell lines expressing MBD3_MBD^MBD2^ or MBD3_Nterm^MBD2^ differentiated normally towards the neuronal lineage, resulting in comparable numbers of live cells and CD24^+^ / CD56^+^ double-positive cells to those observed in WT or to cells rescued with wild type MBD3a (Fig. 4f, g). These results indicate that the methyl-CpG-binding MBD^MBD2^ domain or the MBD2 GR stretch alone, do not influence NuRD complex activity during neuronal differentiation, pointing to a synergy between these two MBD2 parts in the diversification of NuRD complex function.

### Differential nuclear localization of MBD2a- and MBD3a-NuRD complexes

**Fig. 5:**
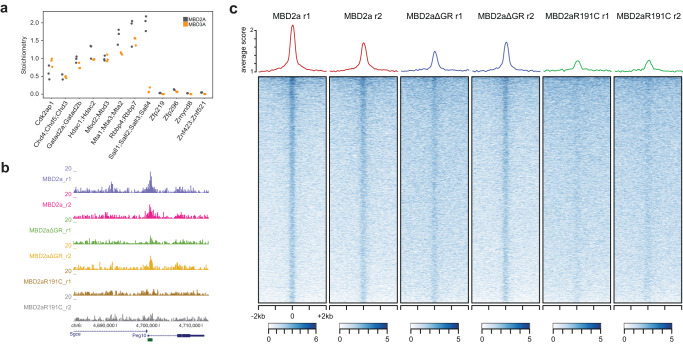
Impact of GR and MBD mutations on MBD2a localization. **a** Stoichiometry determination of NuRD complex members of MS biotin-Co IPs in *Mbd3* KO ESCs stably expressing MBD2a (gray) or MBD3a (orange). The iBAQ value of each protein group is divided by the iBAQ value of MBD2 or MBD3, respectively, then graphed with MBD2/3 set to 1. Proteins with shared peptides are collapsed. n = 3 independent pull-downs. Source data is provided as a Source Data file. **b** MBD2 variant localization to an example chromosomal region. Shown is the enrichment for all MBD2a, MBD2aΔGR, and MBD2R191C proteins at the *Peg10* promoter, which harbors a methylated CpG island (marked as a green bar below the gene profile). MBD protein enrichment is calculated as library-normalized number of tags per 100 bp and two replicates are shown. **c** Heatmaps showing library-normalized ChIP-seq counts in 40 bp windows covering 2 kb upstream and downstream of methylated CpG islands. Average density profiles are indicated on top.

Finally, we investigated if the presence of methyl-CpG-binding domain and GR stretch in MBD2a could influence the NuRD complex composition, in comparison to MBD3. Based on co-immunoprecipitation of biotin-tagged MBD2 and MBD3 versions, followed by antibody detection of core NuRD complex members, we did not observe a strong difference in NuRD complex composition for all tested MBD2 variants, with the exception of MBD2t (Supplementary Fig. 8a). To obtain a more comprehensive and quantitative view on complex stoichiometry, we performed biotin co-immunoprecipitations in *Mbd3* KO ES cells expressing either biotin-tagged MBD2a or MBD3a and identified known NuRD complex members using label-free mass spectrometry (Supplementary Fig. 8b, c). We then calculated the intensity-based absolute quantification (iBAQ) values of the most predominant and statistically significant MBD-interacting proteins in both cell lines, which can be used to estimate the relative abundance. While we observe very similar complex composition between MBD2a-NuRD and MBD3a-NuRD, peptides shared between SALL1-4 proteins show a preferred interaction with the MBD2a-NuRD complex (Fig. 5a).

**Fig. 6:**
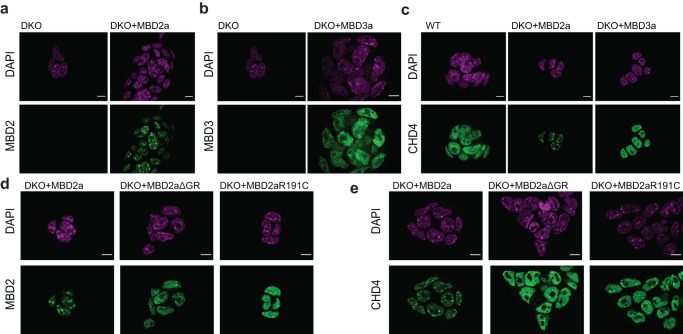
Differential nuclear localization of MBD2a- and MBD3a-NuRD complexes influences neuronal differentiation. **a**, **b** Representative immunofluorescence images indicating MBD2a (**a**) and MBD3a (**b**) localization in *Mbd2,Mbd3*-DKO cells. **c** CHD4 localization in *Mbd2,Mbd3*-DKO cells alone and expressing either MBD2a or MBD3a. **d** MBD2 localization in *Mbd2,Mbd3-*DKO cells stably expressing MBD2a, MBD2aΔGR or MBD2aR191C. **e** CHD4 localization in *Mbd2,Mbd3-*DKO cells stably expressing MBD2a, MBD2aΔGR or MBD2aR191C. DAPI staining (magenta) reveals chromocenters. Similar results were obtained from 2 independent replicates. Scale bars, 5 μm.

SALL4 is an AT-rich DNA binding protein, which is localized to satellite DNA in ESCs^38^. Therefore, we speculated that this difference in NuRD complex composition could indicate differential localization of MBD2a-NuRD and MBD3a-NuRD complexes to methylated pericentromeric satellite repeats. ChIP-seq analysis of genome-wide MBD2a binding indeed indicates a strong affinity for methylated sites in the genome (Fig. 5b, c and Supplementary Fig. 9). This binding preference is strongly reduced for the MBD mutant MBD2aR191C, as previously described^24^, while binding analysis of MBD2aΔGR indicates an intermediate decrease in binding to methylated DNA (Fig. 5b, c and Supplementary Fig. 9). Due to the repetitiveness of satellite repeats, reliable quantitative comparisons between MBD2a, MBD2aΔGR, and MBD2aR191C could not be obtained at these sites. Therefore, we relied on immunofluorescence detection in wild-type, and DKO cells expressing either MBD2a or MBD3a (Fig. 6a–c). These experiments indicate a difference in localization of the MBD2a, MBD3a, and the NuRD complex member CHD4. We observe an increased localization of MBD2a and CHD4 to DAPI-dense chromocenters containing AT-rich Satellite repeats in cells expressing MBD2a (Fig. 6a, c). This was not observed in cells rescued with MBD3a, which showed a more homogenous nuclear staining of MBD3 and CHD4 (Fig. 6b, c). We next investigated the requirement for the MBD domain and GR stretch for the differential nuclear localization of MBD2 and CHD4 localization in cells expressing the MBD2aΔGR or MBD2aR191C variants (Fig. 6d, e). In both cases we observe that the localization of both MBD2 variants and CHD4 to chromocenters is reduced compared with the MBD2a variant, resulting in increased nucleoplasm staining of MBD2 and CHD4 (Fig. 6d, e). The same localization pattern is observed for *Mbd3* KO cells expressing MBD2b (Supplementary Fig. 10a).

This indicates that the MBD domain and the GR stretch of MBD2a cooperate to retain MBD2-NuRD at chromocenters. It remains to be explored how the GR stretch directly contributes to this retention. This part of MBD2a is predicted to be intrinsically disordered, therefore potentially promoting multivalent interactions involved in biomolecular condensate formation. OptoDroplet measurements^39^ based on mCherry-labeled cryptochrome 2 (Cry2) fused to the GR stretch alone, and in combination with the MBD domain, did not reveal increased condensation in comparison to positive controls (Supplementary Fig. 10b–d). Furthermore, the chimeric MBD3 proteins containing the MBD2 MBD domain or the N-terminal part including the GR stretch, did not result in chromocenter localization of CHD4 (Supplementary Fig. 11a), suggesting that both parts (GR stretch and MBD) are required for the observed CHD4 tethering by MBD2a.

## Discussion

Here we provide a systematic dissection of the different MBD2 and MBD3 isoforms and their protein domains during ESC differentiation. In contrast to other tissues where specific MBD isoforms are present, ESCs express all six MBD2/3 variants (MBD2a,b,t and MBD3a,b,c), which can be mutually incorporated into the NuRD complex, ultimately forming distinct assemblies with different functionalities^18,28^. NuRD plays an essential role during lineage commitment regulating both induction and exit from pluripotency and enabling proper lineage differentiation^5,6,18,20,40^. Successful ESC lineage commitment therefore serves as a direct measurement of NuRD complex functionality.

By using a well-defined ESC differentiation model towards NPCs and terminal neurons, we showed that, while MBD3 is critical for ESC lineage commitment, as previously described^18^, this function is independent of its MBD domain. Surprisingly, we found that MBD2 can compensate for the loss of MBD3, leading to the generation of fully differentiated neurons. The functional redundancy was critically dependent on the MBD2 isoform that was used. The MBD2t isoform lacking the C-terminal coiled-coil domain failed to compensate for absence of MBD3, indicating the necessity for MBD2 to integrate in the NuRD complex. A short isoform lacking the N-terminal GR-rich repeat (MBD2b) fully rescued the KO phenotype. Interestingly, the full-length MBD2a isoform also gave rise to fully differentiated neurons, however at a very low rate, suggesting that the presence of the GR repeat is critical for preventing a full rescue in *Mbd3* KO cells. This N-terminal MBD2-specific GR-rich repeat is proposed to influence mCpG-affinity and incorporation of MBD2 to the NuRD complex^11,26^. In addition, the methyl-DNA-binding affinity of the MBD domain in MBD2a also prevented full rescue upon expression in *Mbd3* KO cells, since a point mutation rendering this domain insensitive to methyl-CpG resulted in full differentiation to neuronal cells, with no apparent transcriptional differences to WT cells.

This suggests that the MBD2a isoform may recruit the NuRD complex away from promoters and enhancers, therefore preventing establishment of correct gene expression patterns. Indeed, we observe that MBD2a increases CHD4 localization to chromocenters that contain high levels of methylated Satellite repeat DNA. Additionally, we also show that mutations in the MBD domain or deletion of the GR-rich repeat result in release of CHD4 from chromocenters and uncompromised differentiation of *Mbd3* KO cells, suggesting that these protein structures act synergistically in this tethering. In line with this, the MBD2b isoform that has similar affinity for methylated DNA but lacks the GR-rich repeat leads to a full rescue of the neuronal differentiation block in *Mbd3* KO ESCs, suggesting that indeed both domains, MBD and the GR-rich repeat are needed to cooperate for a “MBD2a-specific” NuRD function. This is further substantiated by our experiments where chimeric MBD3 proteins that carry either the MBD2 MBD domain or GR-rich repeats show full neuronal differentiation and no tethering to chromocenters.

Taken together, the differences observed for the MBD2 isoforms point to a specialized role of these variants in regulating MBD2-NuRD function. Chromatin remodeling complexes often show protein subunit diversity that conveys a specialized function of particular sub-complexes^1,41^. Several studies highlight that NuRD cellular function indeed depends on alternate usage of Mbd2/3, Chd3/4/5 and Mta1/2/3, as MBD2-NuRD but not MBD3-NuRD regulate fetal-hemoglobin switch in adult erythroid cells^42^ and different CHDs subunits regulate neuronal differentiation and migration with a limited protein redundancy^43^. Furthermore, a specific MBD3-GATAD2a NuRD subcomplex was identified to regulate ground-state pluripotency and to block induced pluripotent stem cell formation^44^. The competition between the MBD2 isoforms with MBD3 proteins for other NuRD components results in different assemblies with different functional properties – depending on the MBD variant levels present in the analyzed tissue. This can for example lead to the presence of incomplete NuRD complexes lacking the GATAD2:CHD:CDK2AP1 chromatin remodeling module – as in the case of MBD2t or the newly identified component PWWP2A that replaces MBD2/MBD3 from the NuRD complex (also called NuDe complex)^15–17,23^. This can also result in differential localization of the NuRD complex to genomic sites based on DNA methylation readout by MBD2. While DNA methylation-dependent localization of MBD2 and localization of MBD3 to unmethylated, active regulatory sites have been reported by multiple groups, other NuRD complex members were predominantly found to localize to the latter, with little overlap to DNA-methylated sites^6,24,25,40,45,46^. It remains to be investigated if different MBD2-isoforms lead to the assembly of alternative NuRD (sub-) complexes with distinct genomic localization or display NuRD-independent functions. Taken together, our data highlight a more complex role of MBD2 isoforms and domains in NuRD complex function than previously anticipated.

## Methods

### Cell culture and cell line generation

Mouse embryonic stem cells (HA36CB1, 129×C57BL/6) were cultured as previously described^24^. MBD protein expression constructs were generated in pL1-CAGGS-bio-MCS-polyA-1L^24^. MBD variants without specific domains were achieved by subcloning from initial plasmids using Gibson-Assembly. MBD protein variant expressing cell lines in MBD3 KO ES cells were obtained by RMCE as previously described^24^. Briefly, RMCE constructs were co-transfected with a Cre recombinase expression plasmid (1:0.6 DNA ratio) into RMCE-competent and biotin ligase (BirA)-positive mESCs (HA36CB1)^24^. After 10 days ganciclovir selection (3 mM), individual clones were picked, and construct integration confirmed by PCR and immunoblotting. The MBD2 KO, MBD3 KO and MBD2/3 double knockout (DKO) cell lines were generated by co-transfecting pX330-U6-Chimeric_BB-CBh-hSpCas9 (Addgene 42230) with two sgRNA targeting exon 1 for MBD2 KO cell line (sg1_GACTCCGCCATAGAGCAGGG, sg2_CCCCCCCGGATGGAAGAAGG) and exon 2 and exon 3 for MBD3 KO cell line (sg1_CAACTGGCACGTTACCTGGG, sg2_CACCAACCACCCCAGCAACA), respectively. For the generation of the MBD2/3 DKO cell line the targeting was performed consecutively, first MBD2 single KO cell line # 10 was generated and then exon 2 and exon 3 of MBD3 was targeted (sg1_CAACTGGCACGTTACCTGGG, sg2_CACCAACCACCCCAGCAACA), respectively. pRR-Puro recombination reporter^47^ (Addgene 65853) was co-transfected and 36 hours after transfection cells were treated with 2 µg/ml puromycin for 36 h. Positive KO clones were validated by Sanger sequencing and immunoblotting. Transfections were conducted using Lipofectamine 3000 reagent (Thermo Fisher Scientific) at a 2:1 Lipofectamine/DNA ratio in OptiMEM (Thermo Fisher Scientific). Human U-2 OS cells (authenticated by STR profiling), were cultured as previously described^39^.

### Neuronal differentiation of embryonic stem cells

Neuronal differentiation of embryonic stem cells was performed as previously described^30^. Microscopy images were taken at 20x magnification using an Olympus CKX31 microscope and a Canon Eos 550D Camera. Image contrast was increased for better visualization.

### Embryoid body cultures for FLK1+ mesoderm differentiation

Overall, 24 h prior to the onset of EB cultures, ES cells were transferred on gelatinized plates, using Iscove’s Modified Eagle Medium (IMDM) (Gibco) instead of DMEM. For the generation of embryoid bodie (EB) cultures, ES cells were trypsinized and plated at 10,000 cells/ml in non-adherent 10 cm^2^ petri dishes in EB media containing IMDM supplemented with 1% L-glutamine (Gibco), 10% FBS (Gibco), 0.6% transferrin (Roche, 10652), 50 ug/ml ascorbic acid (Sigma, A4544) and 0.03% monothioglycerol (MTG) (Sigma, M6145). Bmp4 was added from day 0 EB culture and bFGF, activin A and VEGF from day 2.5 (all Peprotech) all at 5 ng/ml. After 5 days in culture EBs were harvested and TrypLE^TM^ Express Enzyme (1×) (Gibco, 12605036) was used to generate a single-cell suspension.

### Flow cytometry

For CD24 and CD56 measurements in neuronal progenitors, single-cell suspensions were obtained from neuronal progenitors after 8 days of differentiation, as previously described^30^. For FLK1 measurements of EB cultures, single-cell suspensions were obtained at day 5 of differentiation. For cell-surface staining, cells were incubated for 30 min at 4 °C with a saturating concentration of anti-CD24a monoclonal antibody (1:200) (eBioscience, Clone M1/69), anti-CD56 monoclonal antibody (1:200) (BD Biosciences, clone 809220) and anti-CD309 (FLK1) monoclonal antibody 1:200 (eBioscience, clone Avas12a1). LIVE/DEAD Fixable Near-IR Dead Cell Stain (L34975, Invitrogen) was used to discriminate cell viability. Samples were acquired using a FACSFortessa (BD Biosciences), and data were analyzed using FlowJo software (version 10.7, Tree Star) and visualized with Prism (version 5.0a).

### Poly-A RNA-sequencing and differential gene expression analysis

Total RNA was isolated from NPCs using the RNeasy Plus mini kit (Qiagen). RNA integrity was measured using a model 2100 Bioanalyzer (Agilent). PolyA-tailed mRNAs were isolated and enriched using NEB Next Poly(A) mRNA Magnetic Isolation Module according to manufacturer’s instructions. Libraries for 1 µg mRNA were prepared using NEB Next UltraTM II Directional RNA Library Prep Kit for Illumina. Sequencing of library pools and read processing were performed on Illumina NovaSeq according to Illumina standards, with 150‐bp single‐end sequencing. Sequencing reads were trimmed using Trim Galore (version 0.6.6) to remove adapter sequenced and aligned using STAR^48^ (version 2.7.7a) using standard options based on the gene transcript annotation gencode.mouse.v1.annotation.gtf (NCBIM37, mm9). Gene counts were obtained using qCount() from QuasR^49^ (version 1.38.0) in R (4.2.3) and differential gene expression was performed using the edgeR package (version 3.40.2) with significance set to p-value < 0.05 and log fold change > I1I^50^. MA and MDS plots were generated with the plotMD() and plotMDS() functions in edgeR. Heatmap representing gene expression changes for selected genes or all genes differentially expressed between WT and MBD3 KO cells were generated using the gplots::heatmap.2() function using log2-transformed, normalized CPM counts (prior.count = 1).

### Chromatin immunoprecipitation and sequencing

Biotin-streptavidin ChIP was performed as previously described in^51^. In brief, 10–20 × 10^6^ cells were fixed for 8 min with 1% formaldehyde at room temperature followed by the addition of glycine to a final concentration of 0.12 M and incubation for 10 min on ice. Cells were harvested and incubated for 10 min in 5 ml 10 mM EDTA, 10 mM TRIS, 0.5 mM EGTA on ice, followed by centrifugation at 680 × g for 5 min. Cells were resuspended in 5 ml buffer containing 0.25% Triton X-100, 1 mM EDTA, 10 mM TRIS, 0.5 mM EGTA, and 200 mM NaCl and incubated for 10 min on ice followed by centrifugation at 680 × g for 5 min. Final cell lysis was performed with 50 mM HEPES, 1 mM EDTA, 1% Triton X-100, 0.1% deoxycholate, 0.2% SDS, and 300 mM NaCl in 1 ml for 1 to 2 h on ice. Cross-linked chromatin was subjected to sonication in a Bioruptor Pico instrument (Diagenode) according to the manufacturer’s instructions. Sonicated chromatin was centrifuged at 12,000 × g for 10 min at 4 °C and supernatant was used for further steps. Streptavidin-M280 magnetic beads were blocked for 1 h with 1% cold fish skin gelatin (Sigma Aldrich) and 100 ng tRNA (Sigma Aldrich) supplemented with protease inhibitor cocktail mix (Roche) and washed twice with buffer 3 with 0.1% SDS and 150 mM NaCl. 150–250 μg chromatin solution was diluted to 0.1% SDS and 150 mM NaCl. Chromatin was then incubated with 30 μl pre-blocked streptavidin-M280 magnetic beads overnight at 4 °C. Beads were washed under rotation for 8 min for each wash step and placed on a magnetic rack for 2 min for exchange of buffers first with two rounds of 2% SDS, high salt buffer (as medium salt buffer, but 0.1% SDS and 500 mM NaCl), DOC buffer (250 mM LiCl, 0.5% NP-40, 0.5% deoxycholate, 1 mM EDTA, 10 mM TRIS), and two rounds of Tris/EDTA buffer. Beads were treated with RNaseA (60 μg, Roche) for 30 min at 37 °C in 1% SDS, 0.1 M NaHCO3, and subsequently proteinase K (60 μg, Roche) for 3 h at 55 °C in 1% SDS, 0.1 M NaHCO3, 10 mM EDTA, 20 mM TRIS, followed by de-cross-linking overnight at 65 °C. DNA was purified with phenol–chloroform extraction and ethanol precipitation. Sequencing libraries were prepared using the NEB-next ChIP-seq library Kit (E62402) following the standard protocols. Samples with different index barcodes were combined at equal molar ratios and sequenced as pools. Sequencing of library pools was performed on Illumina NovaSeq machines according to Illumina standards. Library demultiplexing was performed following Illumina standards.

### ChIP-seq reads processing and data analysis

ChIP-seq samples were filtered for low-quality reads and adapter sequences were removed using Trim Galore (https://github.com/FelixKrueger/TrimGalore) (version 0.6.6). Filtered reads were mapped to the mouse genome (version mm9) using QuasR (version 1.38.0) and the BOWTIE algorithm (version 2.3.5.1) allowing for two mismatches, and only uniquely mapped reads were used (-m 1–best–strata). Genomic annotations are based on the Mus musculus version NCBI37/mm9 from July 2007. CpG island annotation is based on the CpG cluster algorithm^52^ and genome segmentations based on fully DNA methylated regions in ES cells (FMR) were obtained from^53^. CpG islands overlapping with FMR segments were considered as methylated CpG islands. Peaks were called using MACS2 (version 2.1.1.20160309) using the following parameters:–broad -g 1.87e9–broad-cutoff 0.1. Peaks were used to calculate DNA methylation density (mCpG/100 bp) under peak regions and percentage overlaps with genomic segments. Peaks were overlapped with genomic features and coverages were calculated using the following hierarchy: promoters, enhancers, exons, repeats, and introns. Promoters were defined as +/− 1 kb around RefSeq gene TSS, enhancers were defined based on DHS peaks where H3K4me1 was higher than H3K4me3, exons and introns were retrieved based on RefSeq annotations, and repetitive elements using Repeatmasker. Overall, 1 kb intervals were obtained by partitioning the entire genome into 1 kb sized tiles. Intervals overlapping with satellite repeats (Repeatmasker), ENCODE black-listed and low mappability scores (below 0.5) were removed.

### Immunoblotting and biotin/streptavidin pull-downs

Crude nuclear extracts cells were obtained as described in Ref. ^51^. Membranes were blocked with 5% milk or 5% BSA for detection with antibodies or Streptavidin-HRP, respectively. Primary antibodies against MBD2 (1:2000, ab188474, Abcam), MBD3 (1:2000, ab157464, Abcam), MTA2 (1:2500, sc-9447, Santa Cruz Biotechnology), HDAC1 (1:1000, sc-7872, Santa Cruz Biotechnology) or anti-LAMIN B1 (1:1000, sc-374015, Santa Cruz Biotechnology) were used overnight at 4 °C. Protein detection was facilitated using species-specific antibodies conjugated to horseradish peroxidase and Pierce® Peroxidase IHC Detection Kit (Thermo Scientific) or species-specific secondary antibodies with IRDye Fluorescent Dyes (IRDye 800CW goat anti-rabbit IgG, 1:15,000, LI-COR, P/N 925-32211 and IRDye 680RD goat anti-mouse IgG, 1:15,000, LI-COR, P/N 925-68070) were used. Bands were visualized using the Typhoon Biomolecular Imager (Amersham). Biotin detection was performed with Streptavidin-HRP. Molecular weights are indicated by the PageRuler Plus Prestained Protein Ladder (Thermo Fisher Scientific).

For streptavidin-biotin pulldowns, proteins from ESC enriched by crude nuclear fractionation were incubated with 30 μl preblocked (in 0.1% cold fish skin gelatine) Streptavidin-M280 magnetic beads (Invitrogen) in HENG buffer, 150 mM NaCl, at 4 °C overnight. Streptavidin magnetic beads were washed three times each 10 min with HENG buffer, 250 mM NaCl, 0.3% NP40, 1 mM DTT, and protease inhibitors at 4 °C. IPs were resuspended in Laemmli buffer prior to SDS-PAGE and western blotting to PVDF membranes. Membranes were blocked with 5% milk or 5% BSA for detection with antibodies or Streptavidin, respectively. Detection was performed using the Chemidoc MP Imaging System (Biorad). Uncropped and unprocessed scans of all of blots are available as Source Data.

### Nuclear extract preparation and biotin/streptavidin pull-downs for mass spectrometry

Nuclear protein extractions were performed as described previously in Ref. ^54^. In short, cells were harvested with trypsin, washed twice with PBS and spun down for 5 min at 400 x g at 4 °C. Cells were resuspended in five volumes of buffer A (10 mM Hepes KOH pH 7.9, 1.5 mM MgCl2, 10 mM KCl), incubated for 10 min on ice, and then centrifuged for 5 min at 400 × *g* at 4 °C. Cells were resuspended in two volumes of buffer A supplemented with 1xPIC and 0.15% NP40 and transferred to a Dounce homogenizer. After lysis with 40 strokes of a type B (tight) pestle, the suspension was centrifuged for 15 min at 3,200 × *g* at 4 °C. The pellet was washed with PBS and centrifuged again for 5 min at 3,200 × *g* at 4 °C. Then, it was dounced with 10 strokes of a type B pestle in two volumes of buffer C (420 mM NaCl, 20 mM Hepes KOH pH 7.9, 20% (v/v) glycerol, 2 mM MgCl2, 0.2 mM EDTA, 0.1% NP40, 1xPIC, 0.5 mM DTT) and transferred to a new eppendorf tube. This suspension was rotated for 1 h at 4 °C and subsequently centrifuged for 45 min at 20,800 × *g* at 4 °C. The supernatant was collected, aliquoted, snap-frozen in liquid nitrogen and stored at −80 °C.

Label-free biotin/streptavidin pull-downs were performed in triplicate. Per pull-down, 40 μl of M280 streptavidin dynabeads (Invitrogen) was used. Beads were washed twice with buffer C (300 mM NaCl, 20 mM Hepes KOH pH 7.9, 20% (v/v) glycerol, 2 mM MgCl2, 0.2 mM EDTA, 1% NP40, 0.5 mM DTT, 1xPIC). 1 mg of nuclear extract was diluted to a total volume of 400 μl with buffer C with or without NaCl (final concentration 150 mM) and rotated with the beads for 90 min at 4 °C. After beads were washed twice with buffer C (0.5% NP40, 300 mM NaCl), twice with PBS plus 0.5% NP40 and twice with PBS, all supernatant was removed. Beads were then resuspended in 50 μl elution buffer (2 M urea, 100 mM Tris pH 8.2, 10 mM DTT) and incubated for 20 min in a thermoshaker at 1,400 rpm at room temperature. After addition of 50 mM chloroacetamide (CAA), beads were incubated for 10 min at 1,400 rpm at room temperature in the dark. Proteins were then on-bead digested into tryptic peptides by addition of 0.25 μg trypsin and subsequent incubation for 2 h at 1,400 rpm at room temperature. The supernatant was transferred to new tubes and further digested overnight at room temperature with an additional 0.1 μg of trypsin. The digest was stopped by the addition of 10 μl 5% TFA, after which tryptic peptides were purified on C18-StageTips (homemade by the FGCZ)^55^ and stored at 4 °C until use.

### LC-MS/MS measurements and data analysis

Tryptic peptides were eluted from StageTips. Dissolved samples were injected by a Waters M-class UPLC system (Waters AG) operating in trap/elute mode. We have used a Symmetry C18 trap column (5 µm, 0.180 mm × 20 mm, Waters AG) and as separation column a HSS T3 C18 reverse-phase column (1.8 µm, 0.075 mm × 250 mm, Waters AG). The columns were equilibrated with 95% solvent A (0.1% formic acid (FA) in water) and 5% solvent B (0.1% FA in ACN). Trapping of peptides was performed at 15 µl/min for 60 sec and afterward the peptides were eluted using the following linear gradient: 5-35% B in 60 min; 35-98% B in 5 min. The flow rate was constant 0.3 µl/min and the column temperature was controlled at 50 °C. High-accuracy mass spectra were acquired with an Q-Exactive HF mass spectrometer (Thermo Scientific) that was operated in data-dependent acquisition mode. A survey scan was followed by up to 15 MS2 scans. The survey scan was recorded using quadrupole transmission in the mass range of 350-1500 m/z with an AGC target of 3E6, a resolution of 120’000 at 200 m/z and a maximum injection time of 50 ms. All fragment mass spectra were recorded with an AGC target value of 1E5 and a normalized collision energy of 28% and with a resolution of 60’000 at 200 m/z. The maximum injection time of was set to 119 ms and dynamic exclusion was activated for 10 sec.

Peptides were searched with MaxQuant version 1.6.10.43^56^ against the UniProt mouse reference proteome (UP000000589_10090, version November 2020). Settings used were protease cleavage sites K/R, maximum number of missed cleavages 2, fixed modifications Carbamidomethyl (C), variable modifications Oxidation (M); Acetyl (Protein N-term), first search mass tolerance 20 ppm, minimum peptide length 7 amino acids, peptide- and protein-level FDR 0.01, minimum number of unique peptides for protein identification 1, match between runs enabled. As a negative control, a control pull-down with biotin beads and wild-type nuclear extract was used. Statistically enriched proteins were identified by a permutation-based FDR-corrected t-test.

Stoichiometry calculations were performed as described in Ref. ^57^. In brief, to determine the stoichiometry of the identified complexes the relative abundance of the identified interactors as measured by the iBAQ intensities were compared. The background binding level of proteins as measured by the iBAQ intensity in the different control samples was subtracted from the MBD2/3 biotin pulldown iBAQ intensity. Next, these relative abundance values were scaled to the obtained abundance of the bait protein which was set to 1. Volcano plots were produced in R.

### Immunofluorescence

mESC seeded onto 0.2% gelatin-coated coverslips (0.13–0.16 mm – Ted Pella) were fixed with 3.7% formaldehyde in 1x PBS for 10 min at RT. Cells were permeabilized with 0.5% Triton X-100 for 5 min on ice followed by 10 min at RT. Cells were blocked at RT for 1 h with 5% BSA (Sigma-Aldrich) dissolved in PBS-Tween 20 (0.1%). Primary antibodies were diluted in BSA blocking buffer and incubated onto coverslips overnight in a wet chamber at 4 °C (MBD2, 1:1000, ab188474, Abcam; MBD3, 1:1000, ab157464, Abcam; CHD4, 1:1000, ab70469, Abcam). Cells were washed three times with 1× PBS – 0.1% Tween 20/ and once with 1x PBS. Secondary antibodies coupled to Alexa-Fluor 488 or 568 (1: 500; Invitrogen - Molecular probes) were added and coverslips were incubated for 1 h at RT. Cells were washed as above and coverslips were mounted using antifading mounting medium containing DAPI (Vectashield). A Leica Stellaris 5 upright system (University of Zurich Center for Microscopy and Image Analysis) controlled with LASX image acquisition software was used to acquire images using a 63×1.4NA HC PL APO CS2 − 0.14 mm WD oil immersion objective (Leica Microsystems, Germany). Emitted light was detected with 4 Hybrid (Power HyD S) detectors, and one additional detector for transmission (Trans PMT). For supplementary figure 10a, images were acquired with a Zeiss LSM 700 confocal laser scanning microscopy (Biology Image Center, Utrecht University) with a 63×1.4 oil immersion objective (Carl Zeiss, Germany) using 405, 488 nm and 555 nm laser lines. For immunofluorescence of TNs, NPCs were seeded in acid washed, poly-L-ornithine-laminin coated coverslips, fixed and permeabilized as described above. Anti-B-Tubulin III (Tuj1), Sigma T8660, was used on a 1:1000 dilution. Images were obtained using a DeltaVision RT widefield microscope (Cell Microscopy Core, UMC Utrecht, the Netherlands) with a 20x objective (Olympus). Overlap of the emission spectra (bleedthrough) was corrected by automatic and manual adjustments of DAPI, GFP and Texas Red channels and by performing sequential scanning. Images were processed using Fiji (version 2.1.0).

### Cry2 optoDroplet experiments

Cry2 optoDroplet experiments were performed as described previously in Ref. ^39^. Specifically, for optoDroplet quantifications upon blue light exposure, U-2 OS cells were seeded into a 96-well plate (Ibidi µ-plate) and transfected with plasmid DNA for 24 h using TransIT-LT1 (Mirus Bio) according to the manufacturer’s instructions. Afterward, cells were either kept in the dark to serve as negative controls or exposed to 24 cycles of 5 s blue light and 10 s dark in a custom-made blue light box equipped with 8 × 1 W LED lamps with a power of 500 Lm in 10 cm distance to the cells, then fixed in 3% formaldehyde in PBS for 15 min at room temperature and stained with DAPI. Imaging was performed on the Olympus ScanR Screening System (ScanR Image Acquisition 3.01) as previously described in Refs. ^58,59^. The system is equipped with an inverted motorized Olympus IX83 microscope, a motorized stage, IR-laser hardware autofocus, a fast emission filter wheel with one set of bandpass filters for multi-wavelength acquisition (DAPI (ex BP 395/25, em BP 435/26), TRITC (ex BP 550/15, em BP 595/40), and a Hamamatsu ORCA-FLASH 4.0 V2 sCMOS camera (2048 × 2048 pixel, pixel size 6.5 × 6.5 µm) with a 20× UPLSAPO (NA 0.75) air objective. Image information of cell populations was acquired under non-saturating conditions and identical settings were applied to all samples. Image analysis was performed with the Olympus ScanR Image Analysis Software (version 3.0.1), a dynamic background correction was applied, and nuclei segmentation was performed using an integrated intensity-based object detection module based on the DAPI signal. Foci segmentation for optoDroplet detection was performed using an integrated spot-detection module. Downstream analyses were focused on properly detected interphase nuclei containing a 2N-4N DNA content as measured by total and mean DAPI intensities. Expression levels were normalized between all samples and are depicted as arbitrary units. For live-cell analysis, U-2 OS cells were seeded into a 96-well plate (Greiner µclear) and transfected with plasmid DNA 24 h prior to imaging. During time-lapse microscopy, FluoroBrite DMEM supplemented with 10% fetal bovine serum (Gibco) and Glutamax (ThermoFisher Scientific) was used. Time-lapse microscopy was carried out in temperature and CO_2_-controlled conditions (37 °C, 5% CO2) on a GE Healthcare IN Cell Analyzer 2500HS (V7.4) with a PCO sCMOS 16 bit camera (2048 × 2048 pixels, pixel size 6.5 × 6.5 µm) using a CFI Plan Apo Lambda (NA 0.75) 20x air objective at 15 s intervals for 6 min (25 ms ex BP 475/28, em BP 526/52; 100 ms ex BP 575/25, em BP 607.5/19).

### Reporting summary

Further information on research design is available in the Nature Portfolio Reporting Summary linked to this article.

## Supplementary information

Supplementary Information

Reporting Summary

## Data Availability

The data that support this study are available from the corresponding author upon reasonable request. The RNA-seq and ChIP-seq data sets generated in this study have been deposited to NCBI GEO under the accession number GSE199541. The proteomics data sets generated in this study has been deposited to ProteomeXchange under the accession number PXD042407. Source data are provided with this paper.

## Source data

Source Data
