# Supplementary material for: Dissecting the roles of MBD2 isoforms and domains in regulating NuRD complex function during cellular differentiation
Source: Nat Commun. 2023 Jun 29;14:3848. doi: 10.1038/s41467-023-39551-w (PMC10310694; doi:10.1038/s41467-023-39551-w)
Supplement: Supplementary file 1 — Supplementary Information [file 41467_2023_39551_MOESM1_ESM.pdf]

Dissecting the roles of MBD2 isoforms and domains in regulating NuRD complex function during cellular differentiation

Nina Schmolka[1,a], Ino D. Karemaker[1], Richard Cardoso da Silva[1,2], Davide C. Recchia[1,2,3], Vincent Spegg[1,3], Jahnavi Bhaskaran[1,b], Michael Teske[1,3,a], Nathalie P. de Wagenaar[2], Matthias Altmeyer[1], Tuncay Baubec[1,2]

Affiliations:

1) Department of Molecular Mechanisms of Disease, University of Zurich, Zurich, Switzerland.

2) Genome Biology and Epigenetics, Institute of Biodynamics and Biocomplexity, Department of Biology, Faculty of Science, Utrecht University, Utrecht, The Netherlands.

3) Molecular Life Science PhD Program of the Life Science Zurich Graduate School, University of Zurich and ETH Zurich, CH-8057 Zurich, Switzerland

a) Current address: Institute of Experimental Immunology, University of Zurich, Switzerland.

b) Current address: MRC London Institute of Medical Sciences, London, United Kingdom.

Correspondence: t.baubec@uu.nl

Supplementary Figure 1

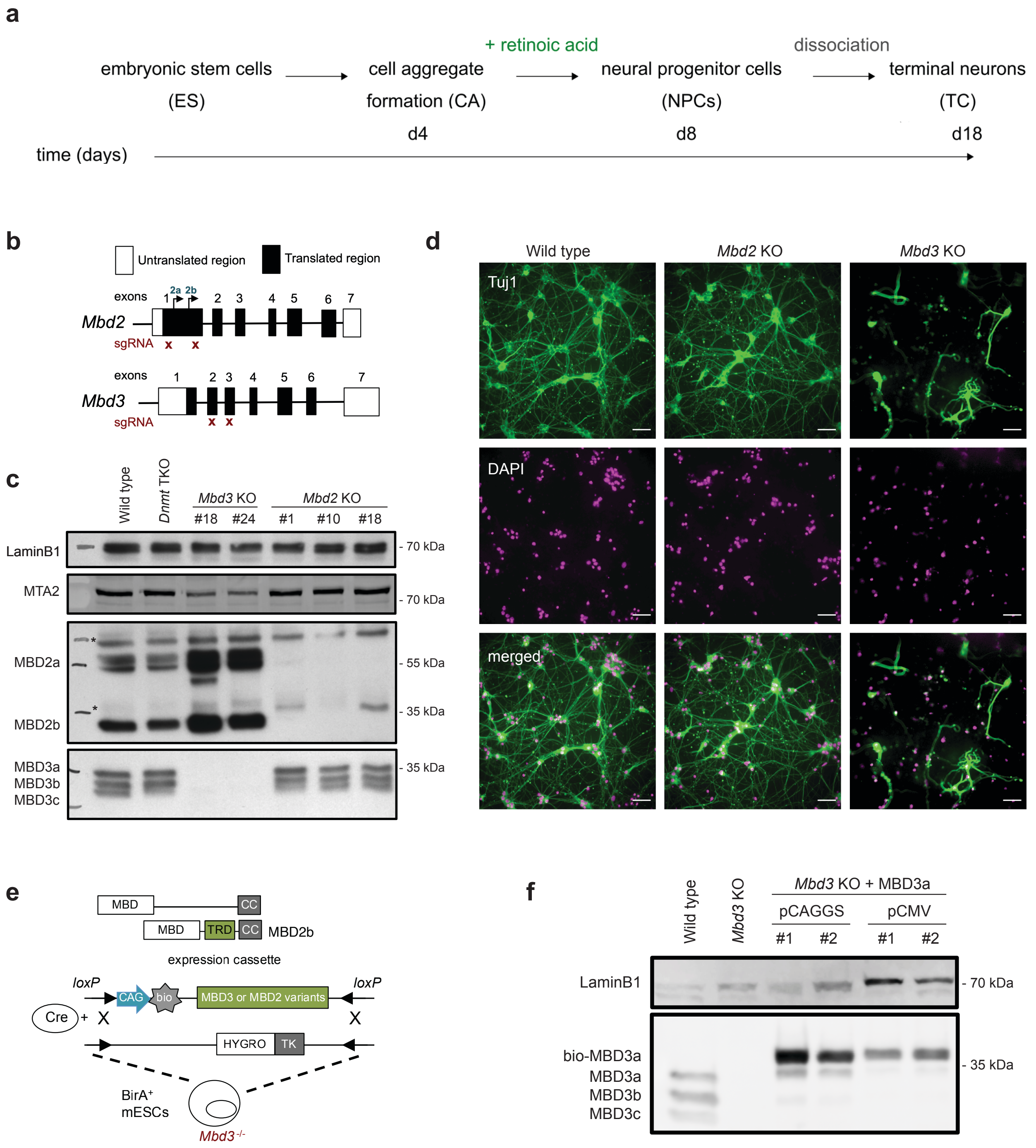

Supplementary Figure 1: a Schematics of the in vitro ESC differentiation protocol towards neuronal progenitors (NPCs) and terminal neurons. **b** CRISPR-Cas9 based targeting strategy to generate *Mbd2* and *Mbd3* knock-out ESC lines. Both KO cell lines were generated with a two single guide (sg) RNA targeting approach. Location of sgRNA binding is indicated by "x" below the gene model. **c** Western blot validation using nuclear extracts from WT and independent *Mbd3* KO and *Mbd2* KO ESC lines probed with antibodies against MBD2 and MBD3. LaminB1 and

MTA2 act as loading controls. Individual MBD2 and MBD3 isoforms are indicated. Asterisks denote unspecific bands. Approximate sizes are indicated in kDa. Experiment was performed once. **d** Representative immunofluorescence staining for neuronal marker Tuj1 (green) in *Mbd2* KO and *Mbd3* KO cell lines after 18 days of neuronal differentiation. DAPI staining is shown in magenta. Scale bar = 50 µm. Experiment was repeated twice. **e** Principle of cell line generation via Cre recombinase mediated cassette exchange (RMCE) and following ganciclovir selection. Biotin-tagged MBD3/MBD2 variant protein cDNAs are targeted to the same genomic location, enabling stable expression and direct comparison in murine ES cells. Triangles: LoxP sites, TK: Thymidne kinase, CAG: CAG promoter, bio: biotin acceptor site. **f** Western blot indicating MBD3 levels in WT, *Mbd3* KO, *Mbd3* KO +MBD3a driven by either CAG or CMV promoters probed with antibodies against MBD3. Two independently derived lines are shown for both constructs. LaminB1 acts as a loading control. Approximate sizes are indicated in kDa.

Supplementary Figure 2

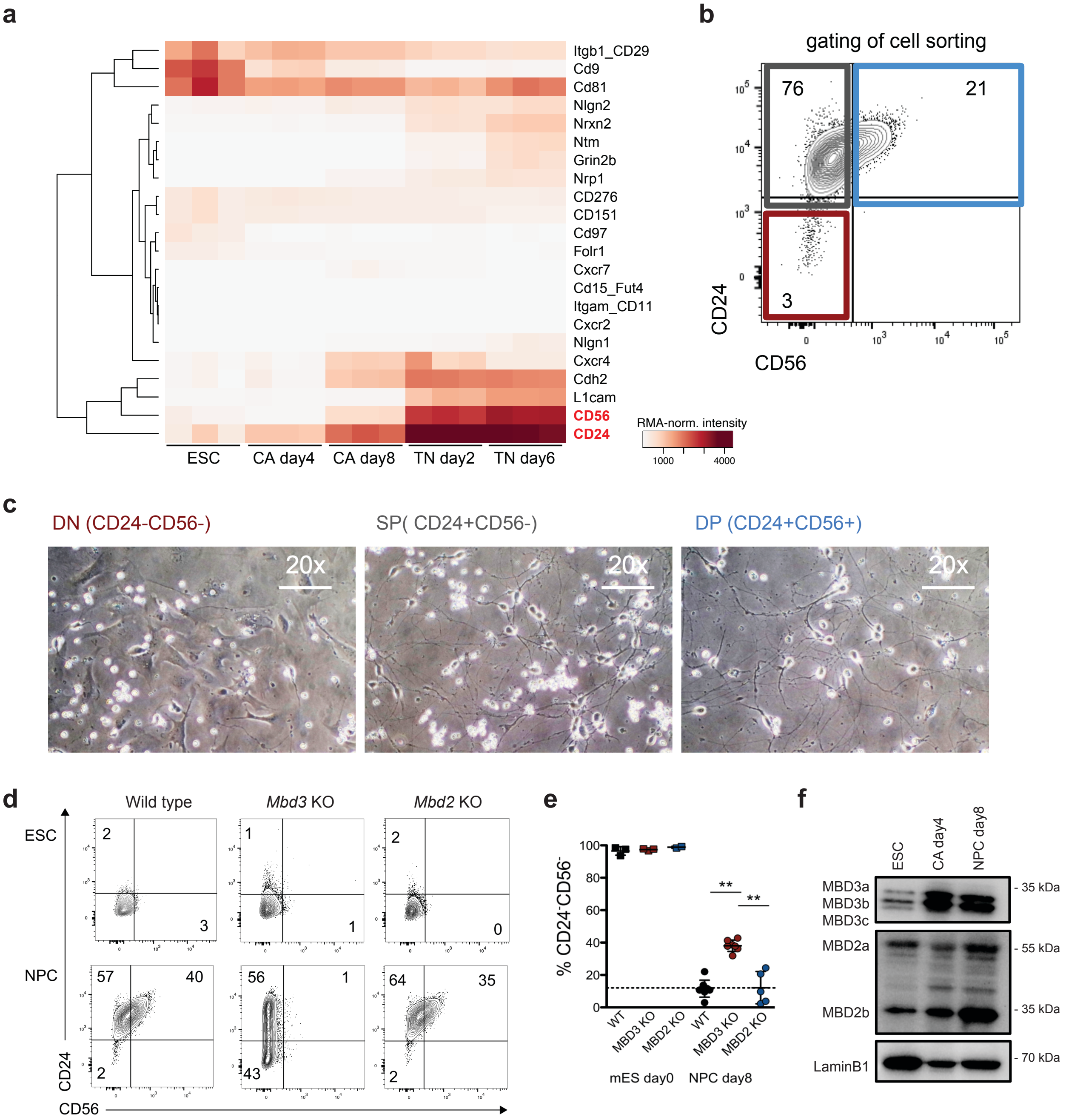

Supplementary Figure 2: a Heat map showing gene expression of selected surface proteins at different stages of neuronal differentiation. Data is shown in triplicates for ESC, CA day4, NPC day8, TN day2 and TN day4. Shown are RMA-normalized microarray intensity values. Source data is provided as a Source Data file. **b** Gating strategy to isolate CD24-CD56- double negative (DN), CD24+ single positive (SP), and CD24+CD56+ double positive (DP) WT NPCs at day8 by FACS. **c** Representative microscopy images of *in vitro* derived neurons from sorted DN, SP, and DP populations shown at 20x magnification. Experiment was performed once. **d** Representative flow cytometry analysis of CD24 and CD56 surface expression in ES (top) and NPC day8

(bottom) of WT, *Mbd3* KO, and *Mbd2* KO cell lines. Numbers in quadrants of flow cytometry plots indicate percentages of cells. **e** Percentage of CD24-CD56- cells in ESC and NPCs day8. Each measurement is obtained from individually generated cell lines. WT to *Mbd3* KO p=0.0014, *Mbd3* KO to *Mbd2* KO p=0.0057. Error bars represent mean +/- SD. p-values were calculated using an unpaired, two-tailed t test (Mann-Whitney). n=2 biological replicates. Source data is provided as a Source Data file. **f** Western blot indicating MBD2 and MBD3 levels in WT ESC, CA day4, and NPC day8. LaminB1 acts as a loading control. Approximate sizes are indicated in kDa. Experiment was repeated three times.

Supplementary Figure 3

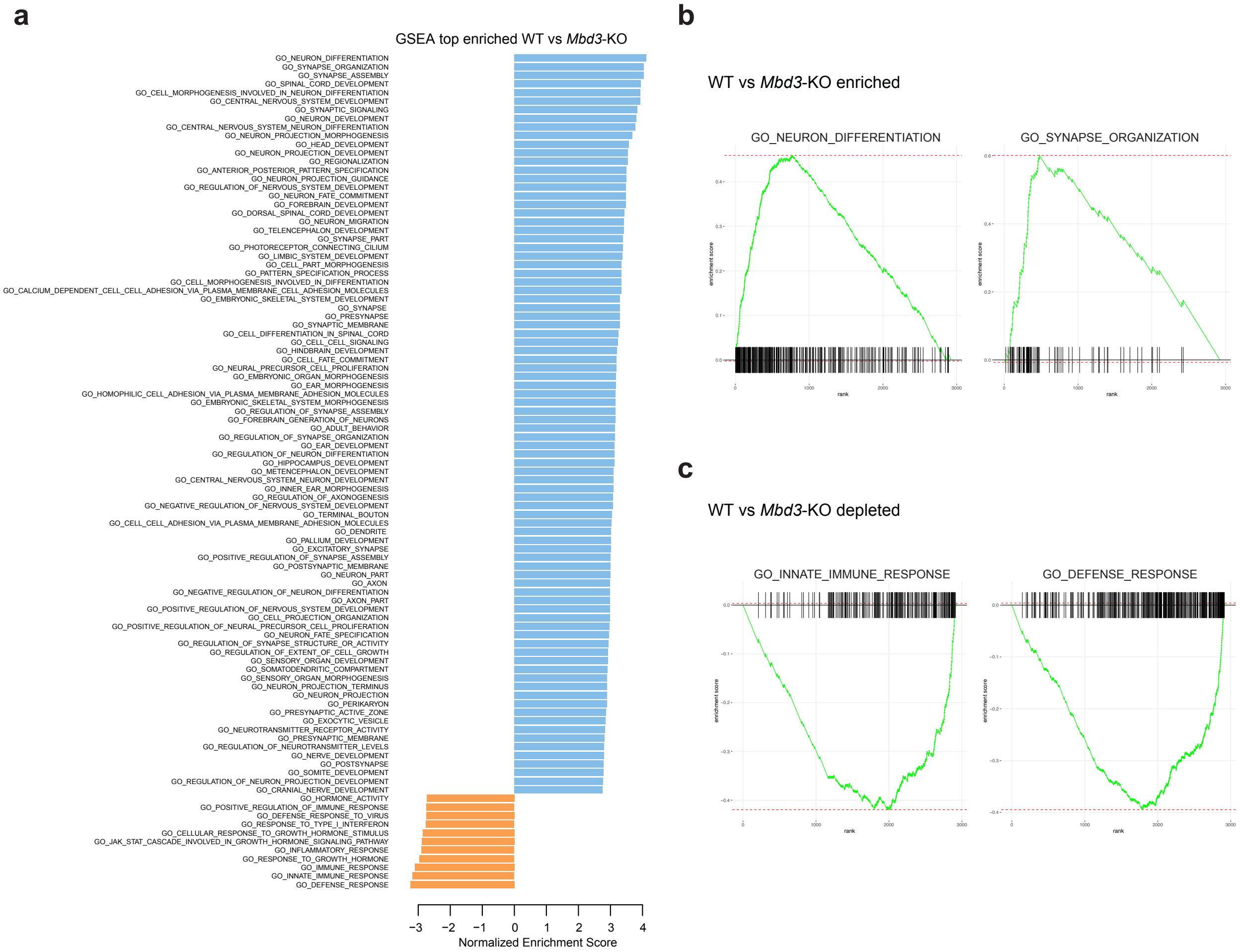

Supplementary Figure 3: a Gene set enrichment analysis (GSEA) of gene ontology (GO) pathways differentially expressed in WT versus *Mbd3* KO NPCs ranked by normalized enrichment score. **b-c** GSEA plots of the two top-most enriched and depleted GO pathways in WT versus *Mbd3* KO NPCs.

Supplementary Figure 4

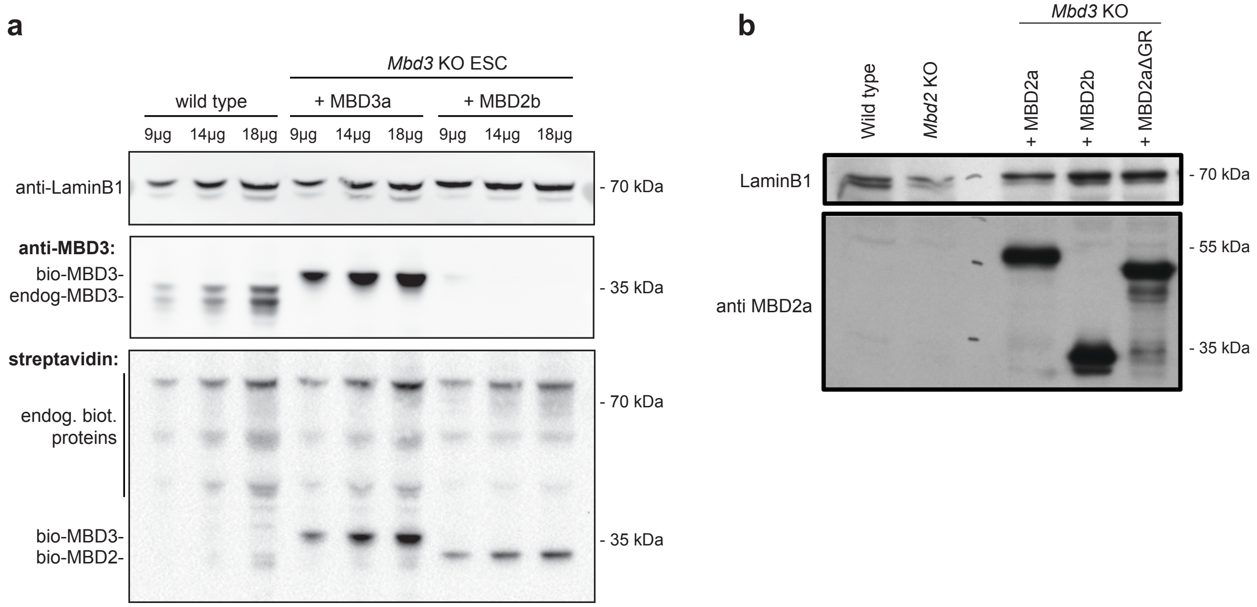

Supplementary Figure 4: a Western blot validation of *Mbd3* KO ESC lines, stably expressing MBD3a or MBD2b, probed with MBD3 and streptavidin-HRP to indicate comparable expression levels of endogenous MBD3 in wild type cells, and biotin-tagged MBD2 and MBD3 from the RMCE site in *Mbd3*-KO cells. Increasing amounts of protein extracts (9µg, 14µg, 18µg) were loaded for better visualisation. LaminB1 acts as a loading control. Approximate sizes are indicated in kDa. Experiment was repeated twice. **b** Western blot validation of *Mbd2* KO, and *Mbd3* KO +MBD2aΔGR, *Mbd3* KO +MBD2b, *Mbd3* KO +MBD2a ESC lines probed with antibodies against MBD2. Detected bands indicate equal expression levels for these different MBD2 constructs. LaminB1 acts as a loading control. Approximate sizes are indicated in kDa. Experiment was performed multiple times.

Supplementary Figure 5

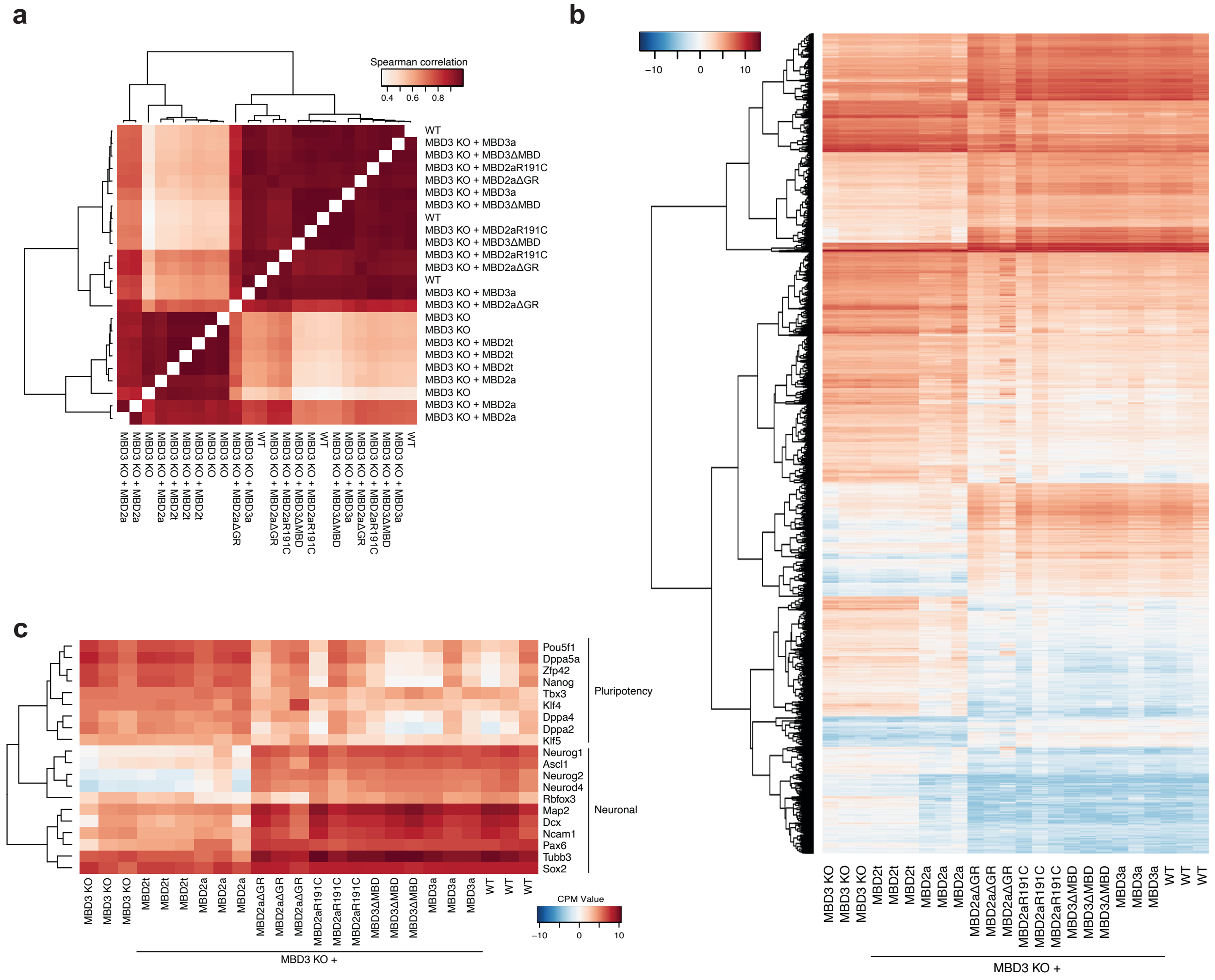

Supplementary Figure 5: a Cross-correlation matrix and unsupervised clustering based on genes differentially expressed between WT and *Mbd3* KO NPCs, indicates similarities in gene expression between the analyzed cell types. Spearman rank correlation is shown. **b** Unsupervised clustering of all differentially expressed genes between WT and *Mbd3* KO NPCs shows their expression in the analyzed samples. Three individually derived clones were analyzed for all indicated cell lines except for *Mbd3* KO +MBD2t where technical replicates of one clone were analyzed. **c** Heatmap indicating gene expression by RNA-seq of pluripotency- associated and neuronal-specific genes in the analyzed samples. Shown in CPM values.

Supplementary Figure 6

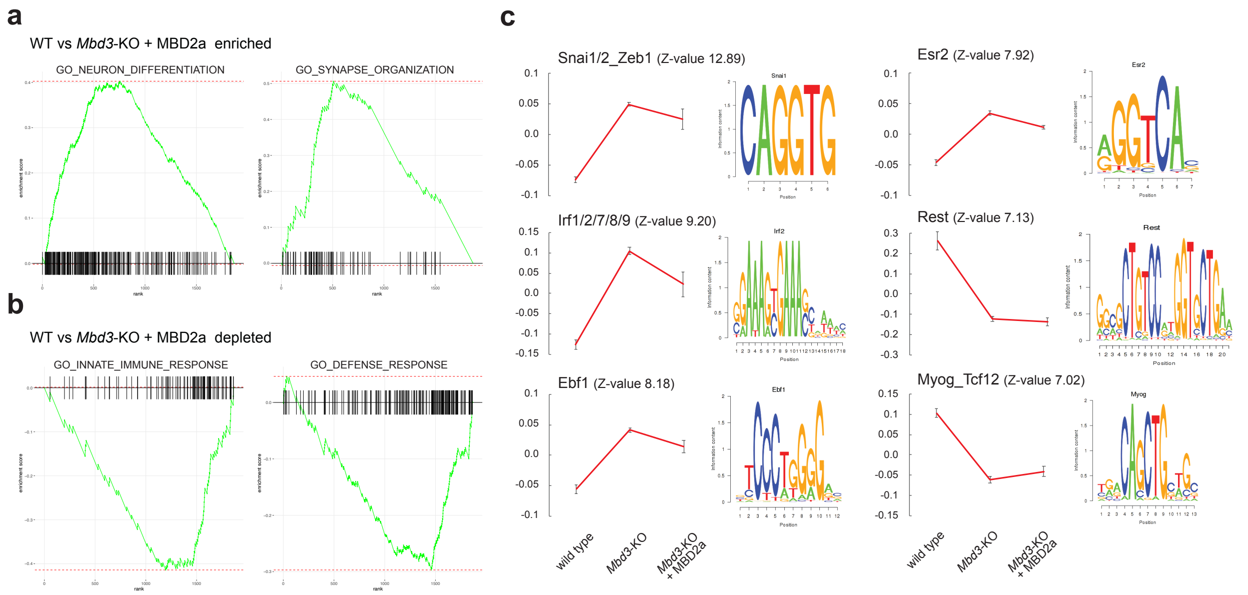

Supplementary Figure 6: GSEA plots of the: **a** two top-most enriched and **b** depleted GO pathways in WT versus *Mbd3* KO + MBD2a NPCs. **c** Shown are the normalized ISMARA activity profiles of the top six transcription factor motifs (red lines, with standard errors) and their respective z-values. Data obtained from three biological replicates of wild type, *Mbd3* KO and *Mbd3* KO +MBD2a are shown. Error bars represent +/- SD. Corresponding sequence logos of TF motifs are shown. Source data is provided as a Source Data file.

Supplementary Figure 7

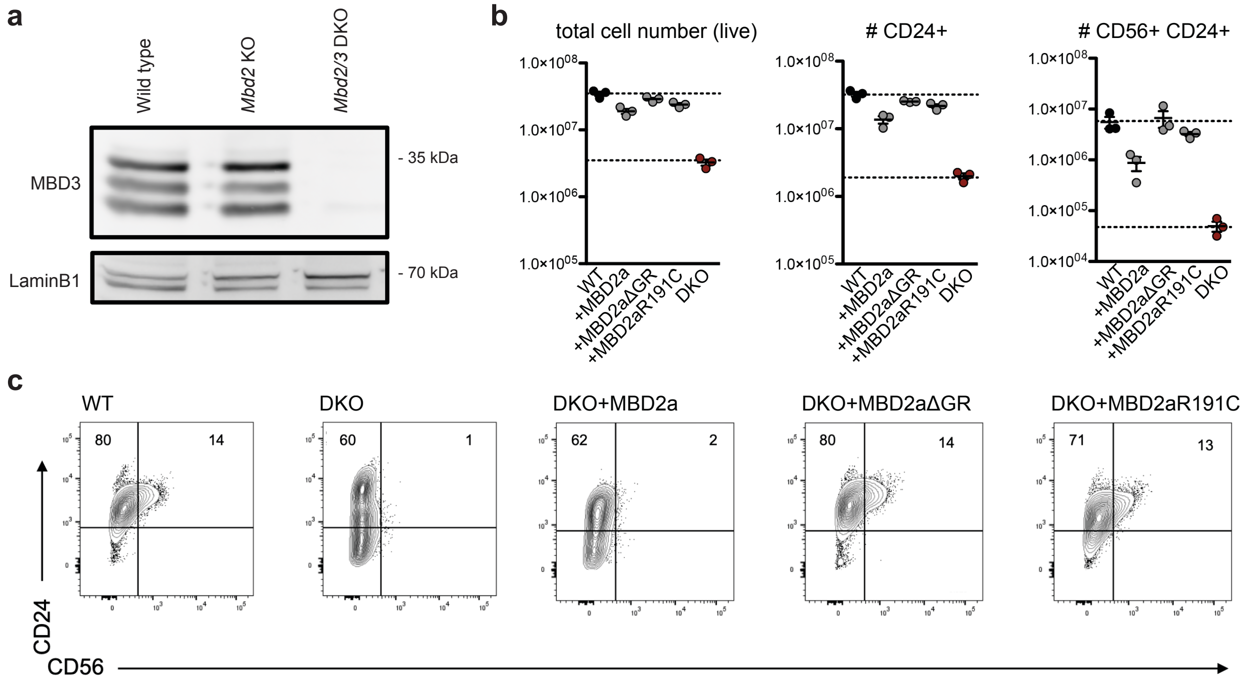

Supplementary Figure 7: a Western blot validation of *Mbd2*, *Mbd3* DKO cell line using nuclear extracts from WT, *Mbd2* KO and *Mbd2/3* DKO ESC line (resulted from sgRNA targeting of *Mbd3* in *Mbd2* KO ESC line) with an antibody against MBD3. LaminB1 was used as loading control. Approximate sizes are indicated in kDa. **b** Flow cytometry analysis indicating (from left to right) the number of live cells, CD24+, and CD24+CD56+ NPC at day8 of neuronal differentiation in WT, *Mbd2/3* DKO or *Mbd2/3* DKO stably expressing MBD2a, MBD2aΔGR or MBD2aR181C. Each data point represents an individual cell line. Error bars represent mean +/- SD. n=1 biological replicate. For each cell line 3 independent clones were analyzed. Source data is provided as a Source Data file. **c** Representative flow cytometry analysis of CD24 and CD56 surface expression in NPC day8 of cell lines indicated in (b). Numbers in quadrants of flow cytometry plots indicate percentages of cells.

Supplementary Figure 8

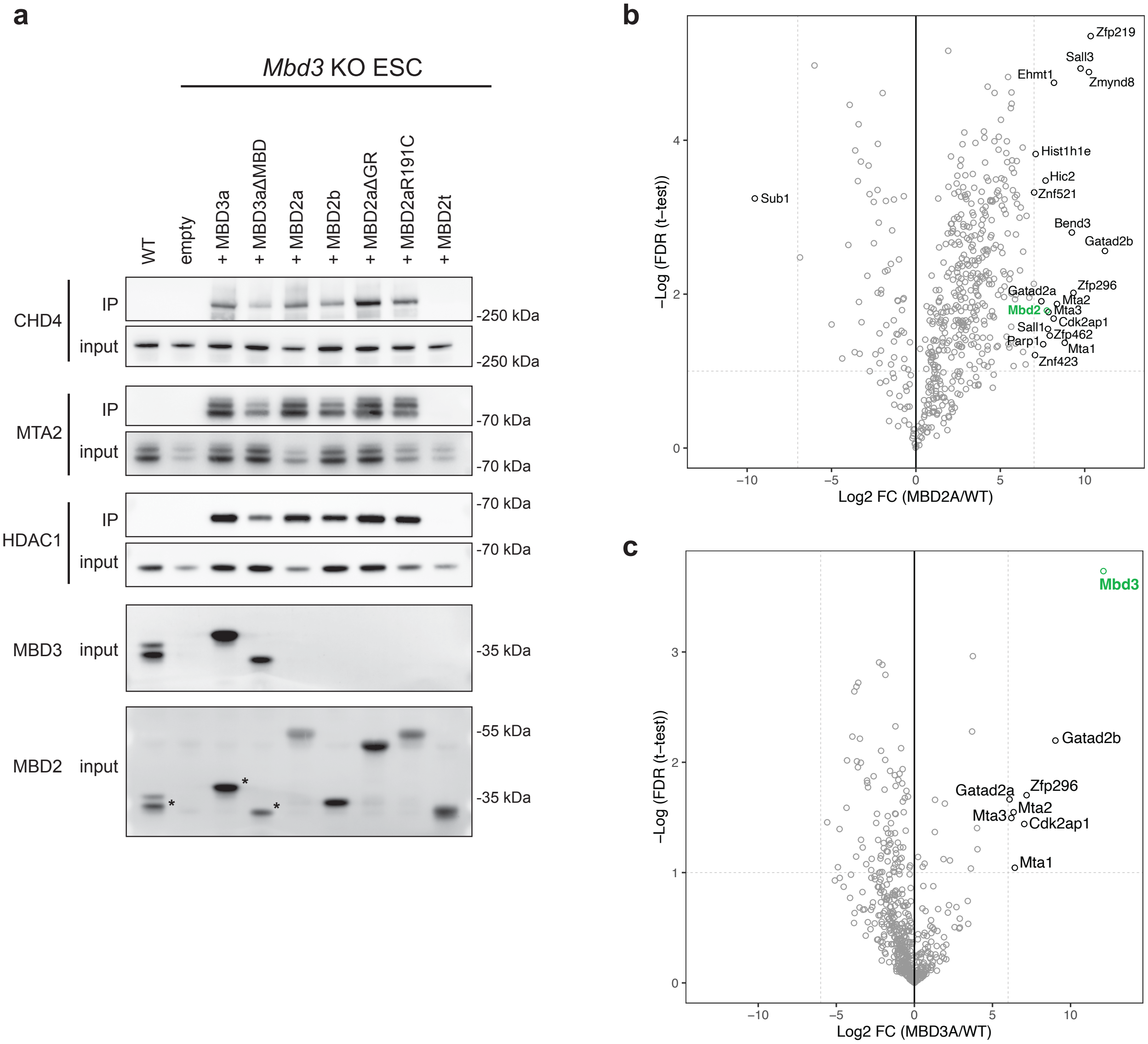

Supplementary Figure 8: a Western blot analysis of immunoprecipitation (IP) of biotin-tagged MBD2 or MBD3 variants in *Mbd3* KO ESC lines and detection of the NuRD complex members: CHD4, MTA2 and HDAC1. Expression levels of biotin-tagged MBD2 or MBD3 proteins are shown for input materials detected with antibodies specific for MBD2 or MBD2, respectively. Asterisks in the MBD2 Western blot are indicating unspecific signals originating from previous MBD3 detection. Approximate sizes are indicated in kDa. Experiment was repeated twice. **b-c** Volcano plots showing MS biotin-Co IP results obtained from *Mbd3* KO ESC stably expressing MBD2a (b) or *Mbd3* KO ESC stably expressing MBD3a (c) in comparison to WT controls. Statistically enriched proteins are indicated (permutation-based FDR-corrected two-tailed t-test: FDR > 1.301, log2 FC > 6 for MBD3a or 7 for MBD2a, n = 3 independent replicates).

Supplementary Figure 9

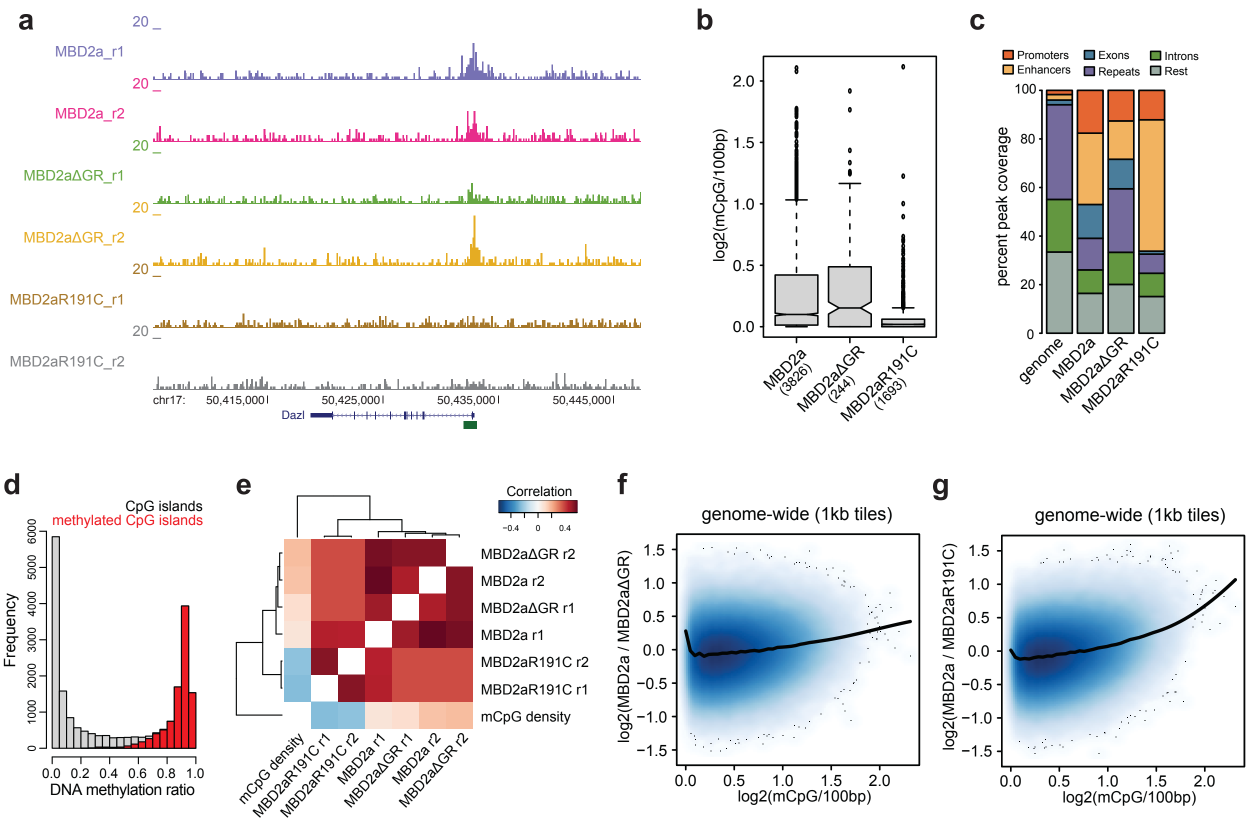

Supplementary Figure 9: a MBD2 variant localization to example chromosomal region. Shown is the enrichment for all MBD2a, MBD2aΔGR and MBD2R191C proteins at the *Dazl* promoter, which harbors a methylated CpG island (marked as a green bar below the gene profile). MBD protein enrichment is calculated as library-normalized number of tags per 100 bp and two replicates are shown. Heat map profiles indicating binding of biotin-tagged MBD2a, MBD2aΔGR, or MBD2aR181C to methylated CpG islands. **b** Box plots showing DNA methylation density under peaks identified for MBD2a, MBD2aΔGR and MBD2R191C. Boxes denote inter-quartile range (IQR), whiskers IQRx1.5. Median value and values outside of IQRx1.5 are indicated. Number of identified peaks are indicated. c Percentage coverage of identified peaks with functional genomic elements. **d** Histogram shows DNA methylation ratio at all CpG islands identified in the mouse genome. CpG islands considered methylated are indicated in red. **e** Cross-correlation of ChIP-seq signals and CpG methylation density at methylated CpG islands. **f-g** Genome-wide scatter plots calculated at 1kb genomic intervals show the impact of ΔGR (f) or R191C (g) on MBD2a binding to DNA methylation density.

Supplementary Figure 10

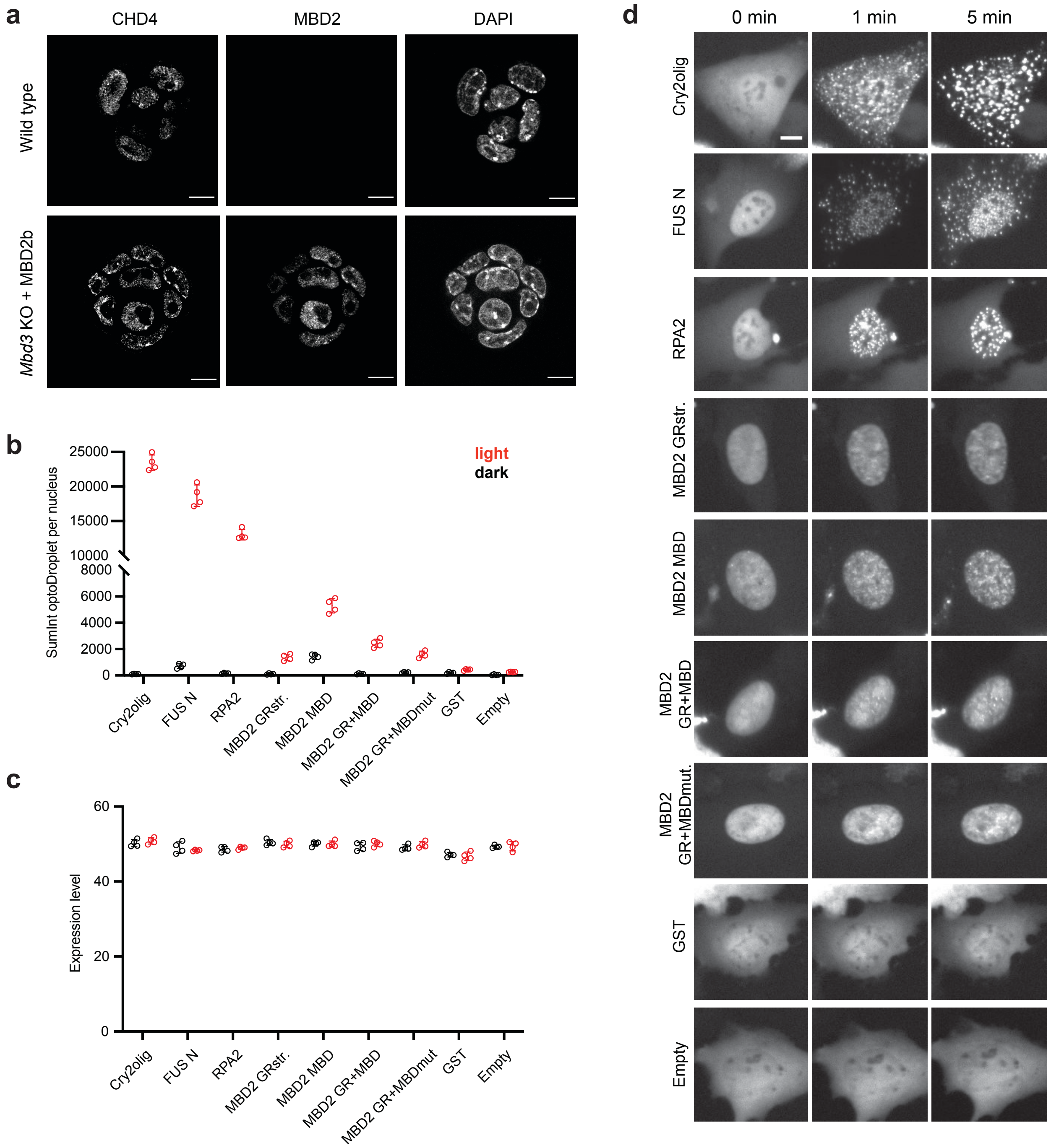

Supplementary Figure 10: a Representative immunofluorescence images of CHD4 and MBD2 in WT and *Mbd3* KO stably expressing MBD2b. Cell lines are indicated above each image. DAPI staining reveals chromocenters. Similar results were obtained from 2 independent replicates. Scale bar = 10 µm. **b** Accumulated optoDroplet intensity per nucleus of different proteins fused to Cry2-mCherry was analyzed with (red) or without (black) optoDroplet induction by blue light for 6min. Averages and standard deviations were calculated from n=4 independent samples per condition (cell number: Cry2oligdark n1=325, n2=400, n3=487, n4=639; Cry2oliglight n1=299,

n2=483, n3=558, n4=507; FUS Ndark n1=370, n2=405, n3=302, n4=408; FUS Nlight n1=404, n2=288, n3=406, n4=563; RPA2dark n1=321, n2=374, n3=477, n4=321; RPA2light n1=386, n2=507, n3=381, n4=370; MBD2 GRstr.dark n1=426, n2=303, n3=339, n4=210; MBD2 GRstr.light n1=415, n2=491, n3=335, n4=432; MBD2 MBDdark n1=432, n2=491, n3=363, n4=387; MBD2 MBDlight n1=565, n2=485, n3=408, n4=431; MBD2 GR+MBDdark n1=279, n2=266, n3=345, n4=419; MBD2 Gr+MBDlight n1=285, n2=428, n3=441, n4=483; MBD2 GR+MBDmutdark n1=379, n2=345, n3=387, n4=230; MBD2 GR+MBDmutlight n1=301, n2=397, n3=301, n4=298; GSTdark n1=448, n2=424, n3=421, n4=483; GSTlight n1=530, n2=665, n3=720, n4=665; Emptydark n1=363, n2=368, n3=391, n4=172; Emptylight n1=456, n2=342, n3=499, n4=503). **c** Nuclear mean intensities of Cry2-mCherry constructs in cells analysed in b. **d** Time-resolved optoDroplet formation of the MBD2 GR stretch, MBD2 MBD domain, MBD2 GR+MBD, and MBD2 GR+MBDmut fused to Cry2-mCherry. Cry2-mCherry fused to FUS N, RPA2, and Cry2-mCherry E490A (Cry2olig) are included as positive controls. Cry2-mCherry-GST and Cry2-mCherry (empty) are included as negative controls. Representative stills from live cell microscopy performed with 15s intervals are shown. Scale bar = 10 µm. n=1 biological replicate. Source data for b-c is provided as a Source Data file.

Supplementary Figure 11

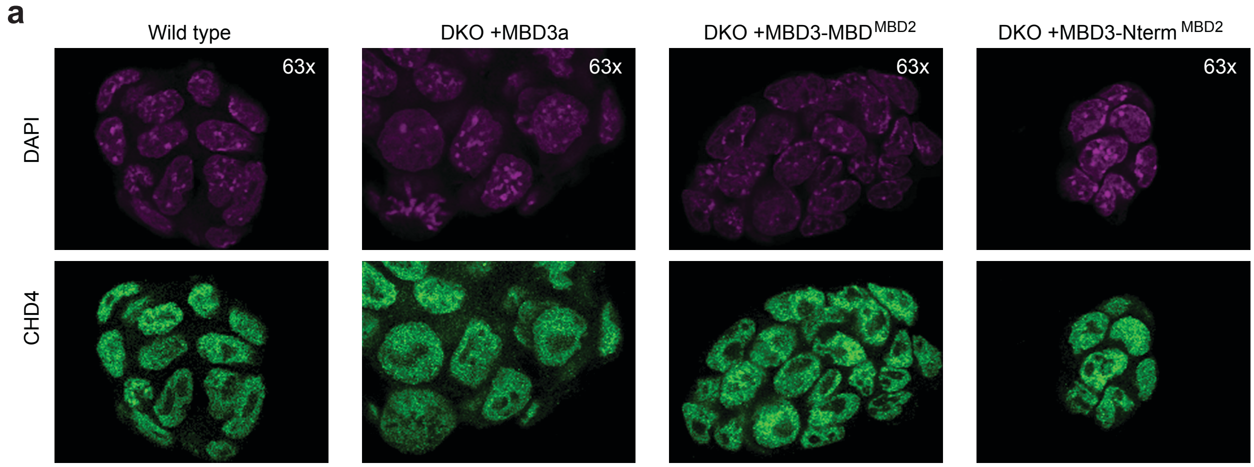

Supplementary Figure 11 a Representative immunofluorescence images of CHD4 in WT or DKO cell lines stably expressing MBD3a, MBD3_MBD^{MBD2} or MBD3_Nterm^{MBD2}. Cell lines are indicated above each image. DAPI staining (Magenta) reveals chromocenters. Similar results were obtained from 2 independent replicates.
