# Supplementary material for: Dissecting the roles of MBD2 isoforms and domains in regulating NuRD complex function during cellular differentiation
Source: Nat Commun. 2023 Jun 29;14:3848. doi: 10.1038/s41467-023-39551-w (PMC10310694; doi:10.1038/s41467-023-39551-w)
Supplement: Supplementary file 2 — Reporting Summary [file 41467_2023_39551_MOESM2_ESM.pdf]

nature portfolio

Corresponding author(s): Tuncay Baubec

Last updated by author(s): May 23, 2023

Reporting Summary

Nature Portfolio wishes to improve the reproducibility of the work that we publish. This form provides structure for consistency and transparency in reporting. For further information on Nature Portfolio policies, see our Editorial Policies and the Editorial Policy Checklist.

Statistics

For all statistical analyses, confirm that the following items are present in the figure legend, table legend, main text, or Methods section.

n/a	Confirmed	
☐	☒	The exact sample size (*n*) for each experimental group/condition, given as a discrete number and unit of measurement
☐	☒	A statement on whether measurements were taken from distinct samples or whether the same sample was measured repeatedly
☐	☒	The statistical test(s) used AND whether they are one- or two-sided<br>*Only common tests should be described solely by name; describe more complex techniques in the Methods section.*
☒	☐	A description of all covariates tested
☐	☒	A description of any assumptions or corrections, such as tests of normality and adjustment for multiple comparisons
☐	☒	A full description of the statistical parameters including central tendency (e.g. means) or other basic estimates (e.g. regression coefficient) AND variation (e.g. standard deviation) or associated estimates of uncertainty (e.g. confidence intervals)
☐	☒	For null hypothesis testing, the test statistic (e.g. *F*, *t*, *r*) with confidence intervals, effect sizes, degrees of freedom and *P* value noted<br>*Give P values as exact values whenever suitable.*
☒	☐	For Bayesian analysis, information on the choice of priors and Markov chain Monte Carlo settings
☒	☐	For hierarchical and complex designs, identification of the appropriate level for tests and full reporting of outcomes
☒	☐	Estimates of effect sizes (e.g. Cohen's *d*, Pearson's *r*), indicating how they were calculated

Our web collection on statistics for biologists contains articles on many of the points above.

Software and code

Policy information about availability of computer code

Data collection	For data collection we used standard operating software installed on Illumina MiSeq/NovaSeq sequencers, BD Biosciences LSR Fortessa cytometer, BD Bioscience FACSAria III cell sorter, and Thermo Scientific Orbitrap Fusion masspectrometers.
Data analysis	For data analysis, the following publicly available tools have been used:<br>FlowJo (10.7)<br>BD FACSDiva (9.1.2)<br>R (4.2.3)<br>trim_galore (0.6.6)<br>EdgeR(3.40.2)<br>MACS2 (2.1.1.20160309)<br>Prism (5.0a)<br>bedtools (2.27.1)<br>MaxQuant (1.6.10.43)<br>Bowtie (2.3.5.1.)<br>STAR(2.7.7a)<br>QuasR(1.38.0)

For manuscripts utilizing custom algorithms or software that are central to the research but not yet described in published literature, software must be made available to editors and reviewers. We strongly encourage code deposition in a community repository (e.g. GitHub). See the Nature Portfolio guidelines for submitting code & software for further information.

Data

Policy information about availability of data

All manuscripts must include a data availability statement. This statement should provide the following information, where applicable:

- Accession codes, unique identifiers, or web links for publicly available datasets
- A description of any restrictions on data availability
- For clinical datasets or third party data, please ensure that the statement adheres to our policy

Newly-generated RNAseq and ChIPseq data sets has been deposited to NCBI GEO under the following accession number GSE199541. Proteomics datasets have been deposited to ProteomeXchange under accession number PXD042407.
published mm9 mouse genome sequence can be found here: https://www.ncbi.nlm.nih.gov/assembly/GCF_000001635.18/

Research involving human participants, their data, or biological material

Policy information about studies with human participants or human data. See also policy information about sex, gender (identity/presentation), and sexual orientation and race, ethnicity and racism.

Reporting on sex and gender	N/A
Reporting on race, ethnicity, or other socially relevant groupings	N/A
Population characteristics	N/A
Recruitment	N/A
Ethics oversight	N/A

Note that full information on the approval of the study protocol must also be provided in the manuscript.

Field-specific reporting

Please select the one below that is the best fit for your research. If you are not sure, read the appropriate sections before making your selection.

☒ Life sciences ☐ Behavioural & social sciences ☐ Ecological, evolutionary & environmental sciences

For a reference copy of the document with all sections, see nature.com/documents/nr-reporting-summary-flat.pdf

Life sciences study design

All studies must disclose on these points even when the disclosure is negative.

Sample size	No statistical method was used to predetermine sample size. In all experiments cell lines were used and we mostly performed n=2 biological replicates with three independently generated cell lines. Results obtained were consistent and did not require larger sample size.
Data exclusions	No data were excluded from the analysis
Replication	All experiments were confirmed by replication at least once with three independent cell lines, except for MBD3KO+MBD2t cell line (only one clone was analysed). The exact handling of replicates is depicted in figure panels or the methods section.
Randomization	Not relevant for the experiments performed here. Samples were allocated to either wild type or mutant and processed in parallel. For MS measurements, block-randomization was applied.
Blinding	Blinding not relevant for this study. All samples were processed through identical analysis pipelines in parallel.

Reporting for specific materials, systems and methods

We require information from authors about some types of materials, experimental systems and methods used in many studies. Here, indicate whether each material, system or method listed is relevant to your study. If you are not sure if a list item applies to your research, read the appropriate section before selecting a response.

Materials & experimental systems

n/a	Involved in the study
☐	☒ Antibodies
☐	☒ Eukaryotic cell lines
☒	☐ Palaeontology and archaeology
☒	☐ Animals and other organisms
☒	☐ Clinical data
☒	☐ Dual use research of concern
☐	☐ Plants

Methods

n/a	Involved in the study
☐	☒ ChIP-seq
☐	☒ Flow cytometry
☐	☐ MRI-based neuroimaging

Antibodies

Antibodies used

The following primary antibodies were used for FACS:
anti-CD24a monoclonal antibody (eBioscience, Ref 48-0242-82, Clone M1/69, Lot 1974978)
anti-CD56 monoclonal antibody (BD Biosciences,Cat 748097, clone: 809220, Lot 9253508)
anti-CD309 (FLK1) monoclonal antibody (eBioscience, Ref 17-5821-81, Clone Avas12a1, Lot 2324710)

The following primary antibodies were used for WB:
anti-LaminB1 monoclonal antibody (Santa Cruz Biotechnology, sc-374015, B-10, Lot J3019)
anti-MBD2 monoclonal antibody (Abcam, ab188474,EPR1836, Lot GR222372-8)
anti-MBD3 monoclonal antibody (Abcam, ab157464, EPR9913, Lot GR117405-18)
anti-MTA2 polyclonal (Santa Cruz Biotechnology, sc-9447, C-20)
anti-CHD4 monoclonal antibody (Abcam, ab70469, 3F2/4, Lot GR3269483-7)
anti-HDAC1 polyclonal (Santa Cruz Biotechnology,sc-7872,H-51, Lot GR3269483-7)

The following primary antibodies were used for IF:
anti-MBD2 monoclonal antibody (Abcam, ab188474,EPR1836, Lot GR222372-8)
anti-MBD3 monoclonal antibody (Abcam, ab157464, EPR9913, Lot GR117405-18)
anti-CHD4 monoclonal antibody (Abcam, ab70469, 3F2/4, Lot GR3269483-7)

The following secondary antibodies were used for WB:
IRDye800CW Goat anti-Rabbit IgG Secondary Antibody (LI-COR, 926-32211)
IRDye 680RD goat anti-mouse IgG Secondary Antibody(LI-COR, 925-68070)

The following secondary antibodies were used for IF:
Goat anti-Mouse IgG (H+L) Cross-Adsorbed Secondary Antibody, Alexa Fluor 568 (Invitrogen, A11004, Lot 2332536)
Goat anti-Mouse IgG (H+L) Cross-Adsorbed Secondary Antibody, Alexa Fluor™ 488 (Invitrogen, A11001, Lot 2465113)
Goat anti-Rabbit IgG (H+L) Highly Cross-Adsorbed Secondary Antibody, Alexa Fluor™ 488 (Invitrogen, A11034, Lot 238003)

Validation

All antibodies are commercial antibodies and validated by the provider and numerous publications.

anti-CD24:
https://www.thermofisher.com/antibody/product/CD24-Antibody-clone-M1-69-Monoclonal/48-0242-82
anti-CD56:
https://www.bdbiosciences.com/en-ca/products/reagents/flow-cytometry-reagents/research-reagents/single-color-antibodies-ruo/BV605-Rat-Anti-Mouse-CD56-(NCAM-1).748097
anti-FLK1:
https://www.thermofisher.com/antibody/product/CD309-FLK1-Antibody-clone-Avas12a1-Monoclonal/17-5821-81
anti LaminB1:
https://www.scbt.com/de/p/lamin-b1-antibody-b-10
anti-MBD2:
https://www.abcam.com/products/primary-antibodies/mbd2-antibody-epr18361-ab188474.html
anti-MBD3:
https://www.abcam.com/products/primary-antibodies/mbd3-antibody-epr9913-chip-grade-ab157464.html
anti-MTA2:
https://www.scbt.com/p/mta2-antibody-c-20
anti-CHD4:
https://www.abcam.com/products/primary-antibodies/chd4-antibody-3f24-ab70469.html
anti-HDAC1:
https://www.scbt.com/p/hdac1-antibody-h-51

Eukaryotic cell lines

Policy information about cell lines and Sex and Gender in Research

Cell line source(s)	murine embryonic stem cells (HA36CB1, 129×C57BL/6) Baubec et al. DOI:10.1016/j.cell.2013.03.011<br>U-2 OS cells: ATCC (HTB-96; RRID:CVCL_0042)
Authentication	mESC:Genotype confirmed by PCR/Sanger sequencing and Western blot.<br>U-2 OS: The parental U-2 OS cell line was authenticated by STR profiling.
Mycoplasma contamination	mESC and U-2 OS cells were tested every 6 weeks for mycoplasma contamination and always scored negative.
Commonly misidentified lines (See ICLAC register)	No commonly misidentified cell lines were used.

Plants

Seed stocks	*Report on the source of all seed stocks or other plant material used. If applicable, state the seed stock centre and catalogue number. If plant specimens were collected from the field, describe the collection location, date and sampling procedures.*
Novel plant genotypes	*Describe the methods by which all novel plant genotypes were produced. This includes those generated by transgenic approaches, gene editing, chemical/radiation-based mutagenesis and hybridization. For transgenic lines, describe the transformation method, the number of independent lines analyzed and the generation upon which experiments were performed. For gene-edited lines, describe the editor used, the endogenous sequence targeted for editing, the targeting guide RNA sequence (if applicable) and how the editor was applied.*
Authentication	*Describe any authentication procedures for each seed stock used or novel genotype generated. Describe any experiments used to assess the effect of a mutation and, where applicable, how potential secondary effects (e.g. second site T-DNA insertions, mosiacism, off-target gene editing) were examined.*

ChIP-seq

Data deposition

☒ Confirm that both raw and final processed data have been deposited in a public database such as GEO.

☒ Confirm that you have deposited or provided access to graph files (e.g. BED files) for the called peaks.

Data access links<br>*May remain private before publication.*	https://www.ncbi.nlm.nih.gov/geo/query/acc.cgi?acc=GSE199541
Files in database submission	ChIP-seq data for MBD2-biotin
Genome browser session (e.g. UCSC)	no longer applicable

Methodology

Replicates	two replicate per clone performed
Sequencing depth	Over 20 Mio reads aligned once after deduplication and filtering of reads with MAPQ >40
Antibodies	no antibodies were used. Biotin/streptavidin pull-down with M280 streptavidin dynabeads
Peak calling parameters	--broad -g 1.87e9 --broad-cutoff 0.1
Data quality	Sequencing quality was assessed using QuasR qQCReport()
Software	trim_galore 0.6.6<br>QuasR 1.38.0<br>MACS2 (2.1.1.20160309)

Flow Cytometry

Plots

Confirm that:

☒ The axis labels state the marker and fluorochrome used (e.g. CD4-FITC).

☒ The axis scales are clearly visible. Include numbers along axes only for bottom left plot of group (a 'group' is an analysis of identical markers).

☒ All plots are contour plots with outliers or pseudocolor plots.

☒ A numerical value for number of cells or percentage (with statistics) is provided.

Methodology

Sample preparation	Cells were harvested by trypsinisation and single-cell suspensions were obtained. For cell-surface staining, cells were incubated for 30min at 4°C with a saturating concentration of anti-CD24a monoclonal antibody (eBioscience, Clone M1/69), anti-CD56 monoclonal antibody (BD Biosciences, clone:809220) and anti-CD309 (FLK1) monoclonal antibody (eBioscience, Clone Avas12a1). LIVE/DEAD Fixable Near-IR Dead Cell Stain (L34975, Invitrogen) was used to discriminate cell viability.
Instrument	LSR Fortessa (BD Biosciences), or FACSAria III cell sorter (BD Biosciences).
Software	Data was acquired with the BD FACSDiva Software (BD Biosciences) and analysed with FlowJo 10.7
Cell population abundance	Purity was assessed for all the samples after sorting.
Gating strategy	Gating was performed based on WT cells gated for single cells, using forward scatter area (FSC-A) vs. side scatter area (SSC-A), followed by FSC-A vs. forward scatter height (FSC-H). Gating for CD24, CD56 and FLK-1 was done with the help of single-staining controls.

☒ Tick this box to confirm that a figure exemplifying the gating strategy is provided in the Supplementary Information.

Magnetic resonance imaging

Experimental design

Design type	*Indicate task or resting state; event-related or block design.*
Design specifications	*Specify the number of blocks, trials or experimental units per session and/or subject, and specify the length of each trial or block (if trials are blocked) and interval between trials.*
Behavioral performance measures	*State number and/or type of variables recorded (e.g. correct button press, response time) and what statistics were used to establish that the subjects were performing the task as expected (e.g. mean, range, and/or standard deviation across subjects).*

Acquisition

Imaging type(s)	*Specify: functional, structural, diffusion, perfusion.*
Field strength	*Specify in Tesla*
Sequence & imaging parameters	*Specify the pulse sequence type (gradient echo, spin echo, etc.), imaging type (EPI, spiral, etc.), field of view, matrix size, slice thickness, orientation and TE/TR/flip angle.*
Area of acquisition	*State whether a whole brain scan was used OR define the area of acquisition, describing how the region was determined.*

Diffusion MRI ☐ Used ☐ Not used

Preprocessing

Preprocessing software	*Provide detail on software version and revision number and on specific parameters (model/functions, brain extraction, segmentation, smoothing kernel size, etc.).*
Normalization	*If data were normalized/standardized, describe the approach(es): specify linear or non-linear and define image types used for transformation OR indicate that data were not normalized and explain rationale for lack of normalization.*
Normalization template	*Describe the template used for normalization/transformation, specifying subject space or group standardized space (e.g. original Talairach, MNI305, ICBM152) OR indicate that the data were not normalized.*

Noise and artifact removal

Describe your procedure(s) for artifact and structured noise removal, specifying motion parameters, tissue signals and physiological signals (heart rate, respiration).

Volume censoring

Define your software and/or method and criteria for volume censoring, and state the extent of such censoring.

Statistical modeling & inference

Model type and settings

Specify type (mass univariate, multivariate, RSA, predictive, etc.) and describe essential details of the model at the first and second levels (e.g. fixed, random or mixed effects; drift or auto-correlation).

Effect(s) tested

Define precise effect in terms of the task or stimulus conditions instead of psychological concepts and indicate whether ANOVA or factorial designs were used.

Specify type of analysis: ☐ Whole brain ☐ ROI-based ☐ Both

Statistic type for inference

(See Eklund et al. 2016)

Specify voxel-wise or cluster-wise and report all relevant parameters for cluster-wise methods.

Correction

Describe the type of correction and how it is obtained for multiple comparisons (e.g. FWE, FDR, permutation or Monte Carlo).

Models & analysis

n/a	Involved in the study
☐	☐ Functional and/or effective connectivity
☐	☐ Graph analysis
☐	☐ Multivariate modeling or predictive analysis

Functional and/or effective connectivity

Report the measures of dependence used and the model details (e.g. Pearson correlation, partial correlation, mutual information).

Graph analysis

Report the dependent variable and connectivity measure, specifying weighted graph or binarized graph, subject- or group-level, and the global and/or node summaries used (e.g. clustering coefficient, efficiency, etc.).

Multivariate modeling and predictive analysis

Specify independent variables, features extraction and dimension reduction, model, training and evaluation metrics.
